# Cerebrovascular Reactivity in Patients With Small Vessel Disease: A Cross-Sectional Study

**DOI:** 10.1161/STROKEAHA.123.042656

**Published:** 2023-10-10

**Authors:** Emilie Sleight, Michael S. Stringer, Una Clancy, Carmen Arteaga, Daniela Jaime Garcia, Will Hewins, Angela C.C. Jochems, Olivia K.L. Hamilton, Cameron Manning, Alasdair G. Morgan, Rachel Locherty, Yajun Cheng, Xiaodi Liu, Junfang Zhang, Iona Hamilton, Charlotte Jardine, Rosalind Brown, Eleni Sakka, Agniete Kampaite, Stewart Wiseman, Maria C. Valdés-Hernández, Francesca M. Chappell, Fergus N. Doubal, Ian Marshall, Michael J. Thrippleton, Joanna M. Wardlaw

**Affiliations:** Centre for Clinical Brain Sciences (E. Sleight, M.S.S., U.C., C.A., D.J.G., W.H., A.C.C.J., O.K.L.H., C.M., A.G.M., R.L., Y.C., X.L., J.Z., R.B., E. Sakka, A.K., S.W., M.C.V.-H., F.M.C., F.N.D., I.M., M.J.T., J.M.W.), University of Edinburgh, United Kingdom.; UK Dementia Research Institute (E. Sleight, M.S.S., U.C., C.A., D.J.G., W.H., A.C.C.J., O.K.L.H., C.M., A.G.M., R.L., Y.C., X.L., J.Z., R.B., E. Sakka, A.K., S.W., M.C.V.-H., F.M.C., F.N.D., I.M., M.J.T., J.M.W.), University of Edinburgh, United Kingdom.; Edinburgh Imaging Facility RIE (I.H., C.J., M.J.T., J.M.W.), University of Edinburgh, United Kingdom.; Department of Neurology, West China Hospital of Sichuan University, Chengdu (Y.C.).; Department of Medicine, University of Hong Kong (X.L.).; Department of Neurology, Shanghai General Hospital, Shanghai Jiao Tong University School of Medicine, China (J.Z.).

**Keywords:** cerebrovascular circulation, cerebrovascular disorders, cognition, cross-sectional studies, humans, magnetic resonance imaging

## Abstract

**BACKGROUND:**

Cerebrovascular reactivity (CVR) is inversely related to white matter hyperintensity severity, a marker of cerebral small vessel disease (SVD). Less is known about the relationship between CVR and other SVD imaging features or cognition. We aimed to investigate these cross-sectional relationships.

**METHODS:**

Between 2018 and 2021 in Edinburgh, we recruited patients presenting with lacunar or cortical ischemic stroke, whom we characterized for SVD features. We measured CVR in subcortical gray matter, normal-appearing white matter, and white matter hyperintensity using 3T magnetic resonance imaging. We assessed cognition using Montreal Cognitive Assessment. Statistical analyses included linear regression models with CVR as outcome, adjusted for age, sex, and vascular risk factors. We reported regression coefficients with 95% CIs.

**RESULTS:**

Of 208 patients, 182 had processable CVR data sets (median age, 68.2 years; 68% men). Although the strength of association depended on tissue type, lower CVR in normal-appearing tissues and white matter hyperintensity was associated with larger white matter hyperintensity volume (B_NAWM_=−0.0073 [95% CI, −0.0133 to −0.0014] %/mm Hg per 10-fold increase in percentage intracranial volume), more lacunes (B_NAWM_=−0.00129 [95% CI, −0.00215 to −0.00043] %/mm Hg per lacune), more microbleeds (B_NAWM_=−0.00083 [95% CI, −0.00130 to −0.00036] %/mm Hg per microbleed), higher deep atrophy score (B_NAWM_=−0.00218 [95% CI, −0.00417 to −0.00020] %/mm Hg per score point increase), higher perivascular space score (B_NAWM_=−0.0034 [95% CI, −0.0066 to −0.0002] %/mm Hg per score point increase in basal ganglia), and higher SVD score (B_NAWM_=−0.0048 [95% CI, −0.0075 to −0.0021] %/mm Hg per score point increase). Lower CVR in normal-appearing tissues was related to lower Montreal Cognitive Assessment without reaching convention statistical significance (B_NAWM_=0.00065 [95% CI, −0.00007 to 0.00137] %/mm Hg per score point increase).

**CONCLUSIONS:**

Lower CVR in patients with SVD was related to more severe SVD burden and worse cognition in this cross-sectional analysis. Longitudinal analysis will help determine whether lower CVR predicts worsening SVD severity or vice versa.

**REGISTRATION:**

URL: https://www.isrctn.com; Unique identifier: ISRCTN12113543.

Cerebral small vessel disease (SVD) is a disorder of the cerebral small vessels causing lacunar ischemic strokes^1^ and vascular cognitive impairment.^2,3^ The associated neuroimaging features observed with magnetic resonance imaging (MRI) are white matter hyperintensities (WMHs), lacunes of presumed vascular origin, microbleeds, enlarged perivascular spaces (PVSs), and recent small subcortical infarcts.^4^ Currently, SVD pathophysiology is unclear; no effective treatments are available.^5^ Therefore, identifying vascular dysfunctions and their relationships to disease features and progression may help develop treatments.^6^

One vascular parameter of interest is cerebrovascular reactivity (CVR), which probes the ability of cerebral blood vessels to dilate in response to increased brain demand for energy and is impaired in patients with SVD.^6–8^ CVR can be obtained by measuring changes in blood oxygen level dependent (BOLD)—an MRI technique sensitive to cerebral blood flow and cerebral blood volume—in response to a vasodilatory stimulus, including carbon dioxide (CO_2_) enriched air.^7,9^

Previous studies investigating CVR in patients with SVD found cross-sectional associations between lower CVR in subcortical gray matter (SGM) and white matter and higher WMH burden.^8,10,11^ One study noted lower CVR in WMH compared with contralateral normal-appearing white matter (NAWM).^12^ Furthermore, SGM and subcortical white matter CVR are associated with higher blood pressure but not with global cerebral blood flow.^8^ White matter CVR is associated with enlarged PVSs in the basal ganglia, increased pulsatility in the venous sinuses, and lower cerebrospinal fluid stroke volume in the foramen magnum.^8^ Global CVR reduction is associated with having more microbleeds but not with the number of lacunes.^13^ Overall, the sample sizes of these studies were relatively small, most of the results have not yet been replicated, and associations of CVR with clinical features such as cognition have not been extensively tested in patients with SVD.

Therefore, we aimed to assess CVR in relation to SVD MRI features at 3T, cognition, and stroke severity in a large cohort of patients with SVD who presented with a minor nondisabling lacunar or cortical ischemic stroke. We hypothesized that lower CVR in normal-appearing tissues and WMH would be associated with more severe SVD imaging features, worse cognition, and stroke severity.

## METHODS

We followed the STROBE (Strengthening the Reporting of Observational Studies in Epidemiology) reporting guidelines.^14^ The data that support the findings of this study will be made available when the study has been completed. In the meantime, they are available from the corresponding author upon reasonable request.

### Patients

Between August 2018 and June 2021, we recruited patients with mild ischemic stroke, either lacunar or mild cortical ischemic stroke, presenting at Edinburgh/Lothian Stroke Services (Mild Stroke Study 3; ISRCTN12113543).^15,16^ Mild stroke was defined as a modified Rankin Scale score ≤2, and stroke diagnosis was undertaken by specialist stroke physicians and neuroradiologists. We excluded patients with MRI contraindications, major neuronal conditions, and severe cardiac and respiratory diseases. All participants gave written informed consent. The Southeast Scotland Regional Ethics Committee approved the study (reference number 18/SS/0044).

Within 3 months of index stroke, all participants underwent MRI. We recorded medical history and vascular risk factors for each patient and measured blood pressure. We assessed global cognition using the Montreal Cognitive Assessment (MoCA). Stroke severity and degree of patient disability were measured using the National Institutes of Health Stroke Scale and modified Rankin Scale.^17^

### MRI Acquisitions

The visit included a 1.5-hour MRI scanning session with breaks for patient comfort. All images were acquired on a 3T MRI scanner (MAGNETOM Prisma; Siemens Healthcare, Erlangen, Germany). We acquired 3-dimensional T_1_-weighted (repetition time [TR]/echo time [TE]/inversion time [TI], 2500/4.37/1100 ms; flip angle, 7°; isotropic resolution, 1.0 mm^3^), 3-dimensional T_2_-weighted (T2W; TR/TE, 3200/408 ms; isotropic resolution, 0.9 mm^3^), 3-dimensional fluid-attenuated inversion recovery (TR/TE/TI, 5000/388/1800 ms; isotropic resolution, 1.0 mm^3^), and 3-dimensional susceptibility-weighted (TR/TE, 28/20 ms; flip angle, 9°; 0.6×0.6×3.0 mm^3^ resolution) images.^15^ We also performed a 2-dimensional gradient-echo echo-planar imaging scan to measure CVR (TR/TE, 1550/30 ms; flip angle, 67°; isotropic resolution, 2.5 mm^3^). Full details of the MRI acquisition protocols including reproducibility can be found in previous works.^7,15,16,18^

During the 12-minute CVR scan, a physician or nurse was present, and we administered medical air and 6% CO_2_-enriched air (CO_2_:O_2_:N_2_, 6%:21%:73%) alternately for 2 and 3 minutes, respectively.^7^ We monitored other physiological parameters: end-tidal CO_2_, end-tidal O_2_, oxygen saturation level, and heart and respiration rates.

### Analysis of MRI Data

Neuroimaging SVD features were assessed using the STRIVE-1 (Standards for ReportIng Vascular Changes on Neuroimaging 1) criteria (Table S1).^4^ We visually assessed WMH, separately in periventricular and deep WM, using Fazekas scores. We visually rated PVS score in the basal ganglia and centrum semiovale.^19^ We also noted the number of lacunes and microbleeds and rated atrophy in deep and superficial brain areas.^20^ We summed Fazekas, PVS, and atrophy scores to get the total Fazekas, PVS, and atrophy scores, respectively. We computed the SVD score, scoring overall SVD severity.^21^

For each individual, all structural images were coregistered to the subject’s T2W image using FSL FLIRT^22,23^ (FMRIB Software Library, FMRIB Analysis Group, Oxford, United Kingdom). Acute stroke lesions were manually segmented on fluid-attenuated inversion recovery images under supervision of an expert neuroradiologist. WMHs were segmented on fluid-attenuated inversion recovery images,^24^ whereas PVS were segmented on T2W images using a previously described computational method.^25,26^ The brain was segmented using the coregistered and combined fluid-attenuated inversion recovery, T_1_-weighted, and T2W images. NAWM masks were generated using an in-house–developed processing pipeline that combines FreeSurfer^27,28^ (https://surfer.nmr.mgh.harvard.edu/) and FSL FAST^29^ outputs. Subcortical structures and ventricles were segmented using FreeSurfer.^27,28^ All masks were checked and rectified manually if needed. WMH and brain volumes were normalized to the intracranial volume and reported in percentage intracranial volume units. PVS volumes were normalized to the volume of the region of interest (ROI) where they were segmented and reported in %ROI volume units.

Regarding CVR data processing, SGM and NAWM masks were eroded in T2W space by 1 mm in all directions to reduce partial volume artifact. To minimize contamination from large blood vessels running along the ventricles, tissue adjacent to the ventricles was excluded using a mask of the ventricles dilated by 5 mm to the left and right and by 4 mm to the anterior, posterior, superior, and inferior directions. We then subtracted the dilated mask from the NAWM and WMH masks. The contribution from other large venous blood vessels was manually removed by comparing all masks to the susceptibility-weighted images. Thereafter, BOLD volumes were temporally realigned. Masks (SGM, NAWM, and WMH) were registered to the mean BOLD space and used to compute the mean BOLD signal in each ROI. We used linear regression to model the mean BOLD signal using a time-shifted end-tidal CO_2_ profile and volume number (to account for linear signal drift) as independent variables.^7,18^ We did not model voxel-wise BOLD signals as this lacks robustness against noise.^18^ The optimal delay per subject and ROI was defined as the time-shift of the end-tidal CO_2_ profile that gave the lowest sum of squared residuals. CVR (in %/mm Hg) was defined as the relative change in BOLD signal per unit change in end-tidal CO_2_. CVR was not assessed in cortical GM due to its thinness, especially in patients with SVD where atrophy including cortical thinning is common, and due to large blood vessels running along the brain surface and causing a large blooming effect thereby contaminating the cortical signal.^7^

### Statistical Analysis

Statistical analyses were conducted using R. We modeled CVR separately in SGM, NAWM, and WMH. Univariate and multivariable linear regressions were conducted using CVR as outcome and SVD features or cognition as independent variables. In the multivariable analyses, we adjusted the models for age, sex, mean arterial pressure, smoking history (current/recent versus ex-smoker for >1 year versus never), diagnosis of hypertension, diabetes, and hypercholesterolemia. We checked for collinearity between variables and verified model assumptions: normality of residuals and heteroscedasticity. To ensure normality of residuals, we transformed WMH volumes using the logarithm to the base-10 function.

We excluded missing data from the relevant analyses. We reported coefficients of the linear regressions with 95% CIs and *P* values. We did not apply corrections for multiple comparisons as we did not use a significance level. We conducted several sensitivity analyses to verify specific technical points (Tables S4 through S9).

## RESULTS

**Figure 1.**
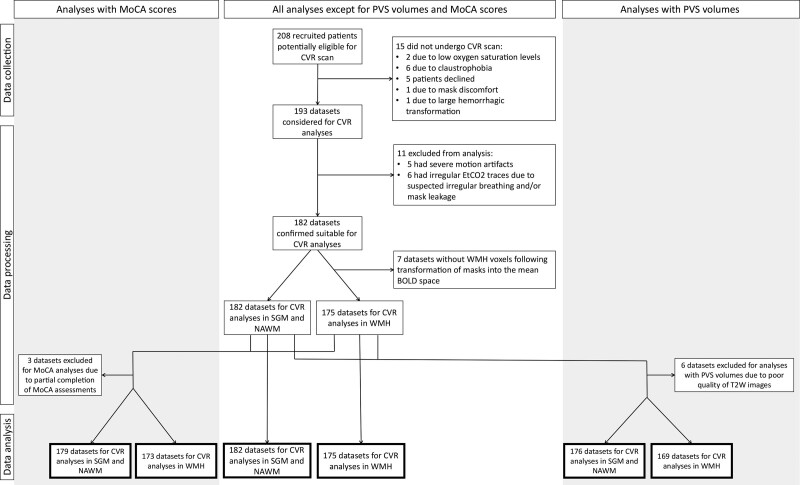
**Flowchart showing data exclusion process before the analysis.** BOLD indicates blood oxygen level dependent; CVR, cerebrovascular reactivity; EtCO2, end-tidal CO_2_; MoCA, Montreal Cognitive Assessment; NAWM, normal-appearing white matter; PVS, perivascular space; SGM, subcortical gray matter; T2W, T2 weighted; and WMH, white matter hyperintensity.

We recruited 208 patients of whom 15 did not undergo CVR (Figure 1). We included 182 of 193 data sets in the analysis (median age, 68.2 years; 68% men; Table 1). Reasons to exclude 11 data sets are given in Figure 1. Of the remaining 182 data sets, 7 patients did not have WMH voxels following mask registration into the mean BOLD space, thus resulting in 175 data sets specifically for WMH CVR analyses. PVS volumes could not be computed in 6 of 182 data sets due to poor quality of T2W images. Full MoCA assessment was not available for 3 of 182 subjects.

**Table 1.**
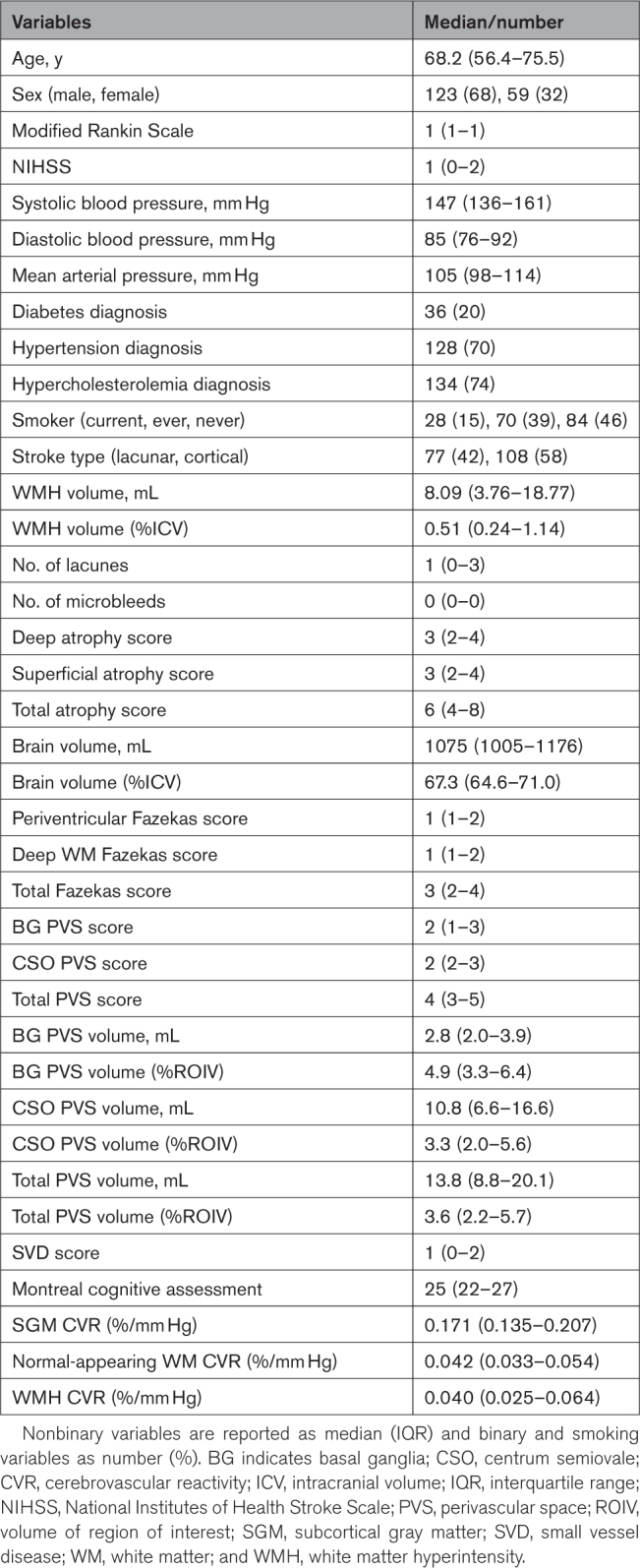
Population Characteristics

CVR was similar in WMH and NAWM (mean inter-region difference, 0.00206 [95% CI, −0.00379 to 0.00791] %/mm Hg) and highest in SGM (SGM−NAWM CVR difference, 0.128 [0.121–0.134] %/mm Hg; Table 1).

**Figure 2.**
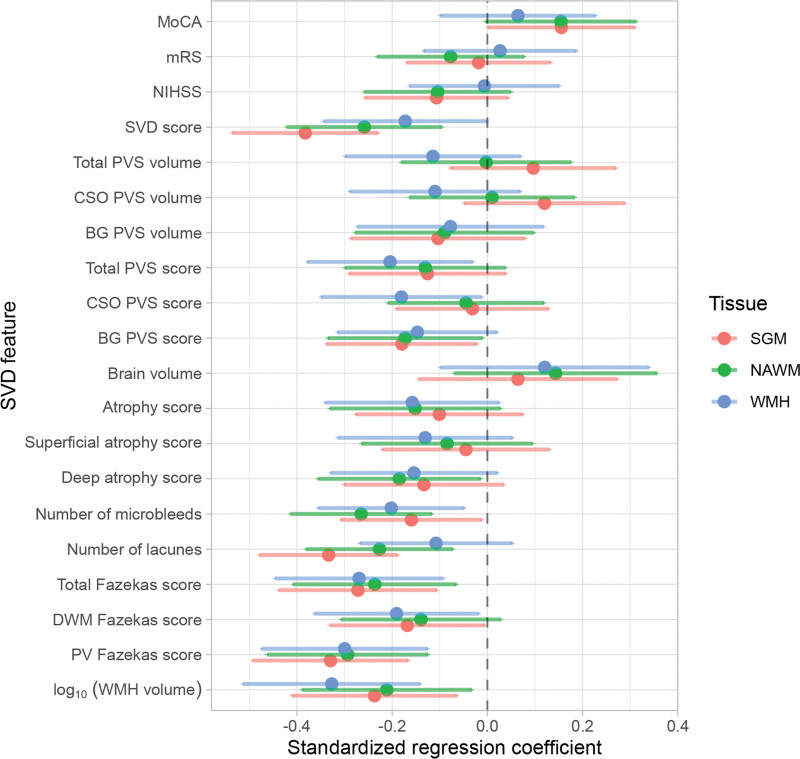
**Standardized regression coefficients between small vessel disease (SVD) features and CVR in subcortical gray matter (SGM; pink), normal-appearing white matter (NAWM; green), and white matter hyperintensity (WMH; blue).** The dots represent the mean standardized coefficients and the horizontal lines, the associated 95% CIs. The vertical dashed line emphasizes a zero-valued coefficient. Coefficients to left of zero line indicate association with lower CVR. BG indicates basal ganglia; CSO, centrum semiovale; DWM, deep white matter; MoCA, Montreal Cognitive Assessment; mRS, modified Rankin Scale; NIHSS, National Institutes of Health Stroke Scale; PV, periventricular; and PVS, perivascular space.

**Figure 3.**
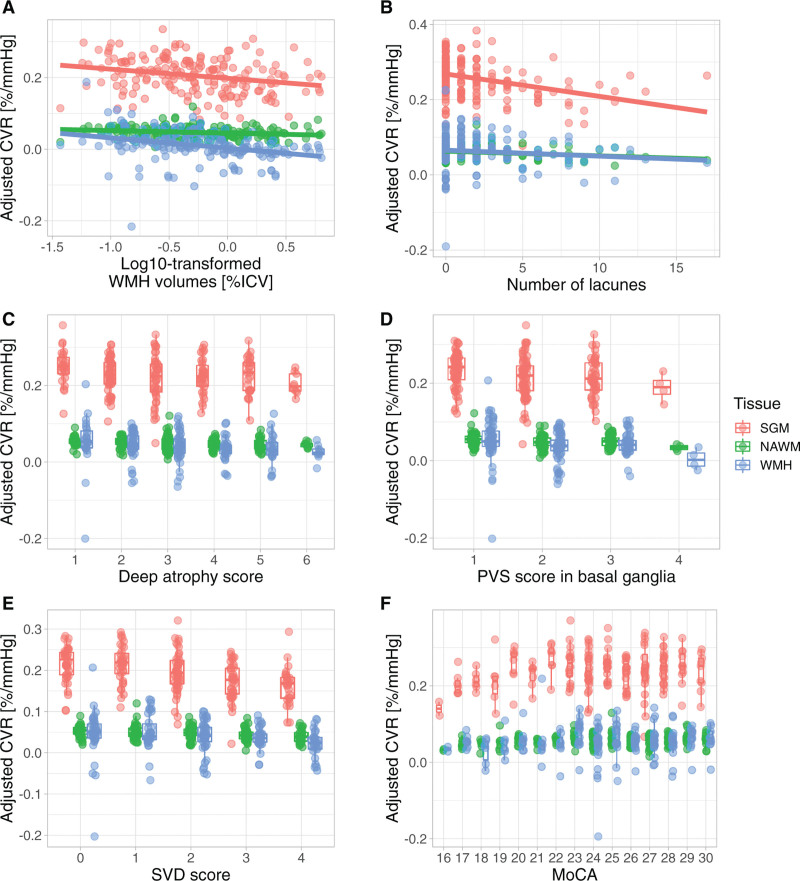
**Relationships between adjusted cerebrovascular reactivity (CVR), small vessel disease (SVD) features, and cognition.** CVR was adjusted for age, sex, and vascular risk factors. The results are shown for adjusted CVR in subcortical gray matter (SGM; pink), normal-appearing white matter (NAWM; green), and white matter hyperintensity (WMH; blue) as a function of (**A**) WMH volume, (**B**) number of lacunes, (**C**) deep atrophy score, (**D**) perivascular space (PVS) score in the basal ganglia, (**E**) SVD score, and (**F**) Montreal Cognitive Assessment (MoCA) score. In **A** and **B**, the regression lines are shown.

Regression coefficients are reported in Table 2 and illustrated in Figures 2 and 3. Lower CVR in most tissues was associated with greater WMH volumes, higher Fazekas scores, more microbleeds, more lacunes, and higher SVD scores, although relationships between WMH CVR and lacunes and between NAWM CVR and deep WM Fazekas scores were not conventionally significant. Lower NAWM CVR was associated with higher deep atrophy scores, with a similar relationship for SGM and WMH CVR. We found an association between lower CVR in normal-appearing tissues and higher basal ganglia PVS scores, with a similar direction of effect for WMH CVR. Moreover, lower WMH CVR was associated with higher centrum semiovale and total PVS scores. There was a general direction of lower CVR in normal-appearing tissues and lower MoCA scores, although not conventionally significant. We did not find associations between CVR, brain volumes, National Institutes of Health Stroke Scale, and modified Rankin Scale scores.

**Table 2.**
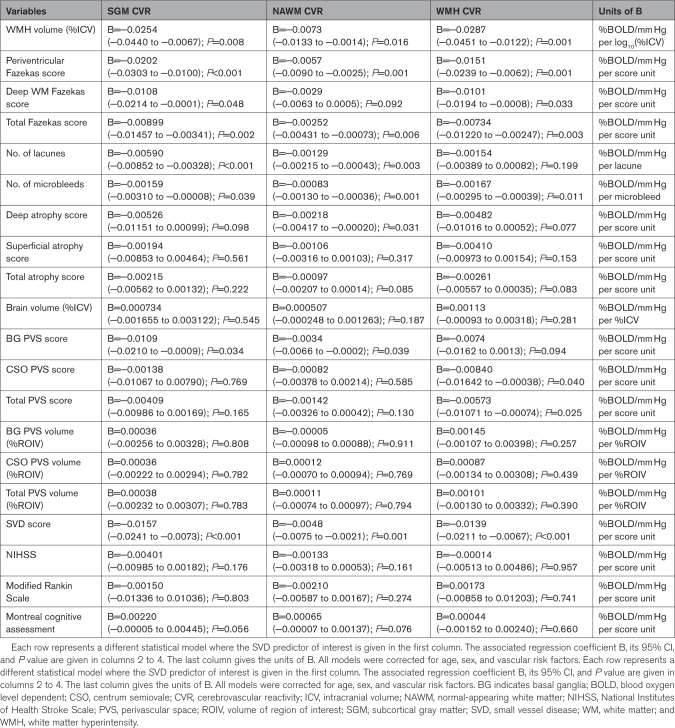
Adjusted Analyses

## DISCUSSION

We investigated how CVR relates to a comprehensive set of SVD features, as well as to cognitive impairment and stroke severity. In this largest study of CVR in SVD to date, CVR was lower in patients with more severe SVD even in normal-appearing tissues, and in association with different SVD features, although the strength of association varied across tissue and lesion types. CVR in normal-appearing tissues and MoCA scores were positively related, although the existence of effect did not reach conventional significance. These relationships were independent of age, sex, and vascular risk factors. As SVD-related tissue damage accumulates over time,^30^ regions with low CVR could be at risk of deteriorating. Indeed, a previous study (n=45) found that CVR in NAWM that progressed into WMH after 1 year was lower than in contralateral NAWM.^31^ Future studies should confirm this.

The relationship between lower CVR and higher WMH burden is consistent between WMH volumes and visual scores. Such relationships have been found in previous studies in older subjects with WMH,^12,31–34^ patients with Alzheimer disease,^35^ and SVD patients with mild stroke.^8^ The effect sizes are similar to those from a previous study.^10^ Overall, the sample size of the current study is larger (n=182 versus n=10–75), thereby making the finding much more robust.

Lower CVR in most ROIs was associated with more lacunes and microbleeds. The coefficient between CVR in WMH and number of lacunes did not reach conventional statistical significance (*P*<0.05), although the direction of effect is biologically plausible. Two previous studies also investigated those relationships but found no associations between CVR and number of lacunes.^8,13^ Results for number of microbleeds differed: 1 study found CVR impairment related to more microbleeds^13^ and the other found no associations.^8^ However, the 2 studies had much smaller sample sizes (n=49–53), data were acquired at different field strengths (1.5T and 7T), and other brain regions were considered for CVR computation.

Lower NAWM CVR was associated with higher deep atrophy score, whereas other relationships between CVR and brain atrophy did not pass the *P*<0.05 threshold. However, based on the coefficient and its 95% CIs, one could argue about the existence of an association between lower WMH CVR and higher deep atrophy scores. A previous study^8^ found no associations between CVR and atrophy, possibly due to smaller sample size (n=53).

We found an association between lower CVR in all ROIs and higher basal ganglia PVS score with lower confidence in the existence of an effect in the case of CVR in WMH. On the contrary, we found associations between WMH CVR and centrum semiovale or total PVS scores. Different relationships with CVR were found when using PVS scores and volumes: whereas scores reflect only a count, volumes will also be influenced by PVS size. Moreover, scores could be limited by floor and ceiling effects.^36^ Previous studies have found that lower CVR^8,37^ and higher vascular pulsatility^38^ are associated with enlarged PVS. Although currently under debate, lower CVR and higher vascular pulsatility could be linked to vascular stiffness, which itself could induce stagnation of interstitial fluid, thereby providing a link between the brain’s waste clearance and vascular systems.^6,39^

We also found an association between lower CVR in all ROIs and higher SVD score in agreement with a previous study.^8^ Therefore, CVR could be a marker reflecting overall SVD severity and should be considered for future clinical studies of SVD.

CVR impairment in normal-appearing tissues was related to worse cognition, although the results did not reach conventional statistical significance. However, this could have been mediated by WMH burden. There were no associations between CVR and stroke outcome or severity, possibly because both were mild. One previous study also reported no associations between CVR and stroke severity or dependency, though its sample size was smaller.^8^ Previous studies on Alzheimer disease have found lower CVR compared with healthy volunteers but did not report on the relationship between CVR and cognition directly.^35,40^

This work has multiple strengths. We used a reproducible CVR experiment optimized for SVD research.^7,10,18^ Visual assessments of SVD features were systematic, comprehensive, and supervised by expert neuroradiologists, and statistical analyses were verified by a professional statistician. Image analysis used pipelines designed and tested in vascular disease. Finally, this is the largest study to date to have assessed CVR impairment in SVD.

There are also some limitations. First, the BOLD contrast is sensitive to cerebral blood flow but also to cerebral blood volume, oxygen extraction fraction, oxygen consumption, hematocrit, and vessel morphology, thereby hindering the interpretation of BOLD signal changes. CVR was not assessed in cortical GM due to associated technical challenges, although this would be relevant in future work. Due to limited repeatability,^18^ CVR delay was not investigated in this study. This analysis only included SVD patients with lacunar or cortical stroke; therefore, the associations found could differ in other forms of SVD. More men were recruited than women, reflecting men excess in small vessel stroke.^41^ The population had mild stroke, but patients with more severe stroke would not be able to tolerate long scans. Moreover, we only used MoCA to reflect cognition, whereas other metrics could be investigated, for example, trail making A and B test.^42^ Lastly, this is a cross-sectional study; therefore, the relationships found are not causal.

Overall, lower CVR in WMH, NAWM, and SGM was associated with SVD burden in patients with mild ischemic stroke and SVD. The strength of association depended on the tissue and SVD feature type. Further research is needed to understand how CVR impairment relates to the progression of SVD lesions.

## ARTICLE INFORMATION

### Acknowledgments

The authors thank the participants, radiographers, and professional support staff for their contribution to this work. The present study is based on Chapter 6 of the doctoral thesis of Dr Sleight conducted at the University of Edinburgh. Dr Sleight prepared the manuscript, contributed to data collection and processing, and did the formal analysis and interpretation of data. Drs Wardlaw, Clancy, Thrippleton, Marshall, and Doubal designed the study. Dr Clancy, Dr Arteaga, D.J. Garcia, and W. Hewins participated in patient recruitment. Dr Stringer, Dr Clancy, Dr Arteaga, D.J. Garcia, W. Hewins, A.C.C. Jochems, Dr Hamilton, Dr Manning, Dr Morgan, R. Locherty, Dr Cheng, Dr Liu, Dr Zhang, I. Hamilton, C. Jardine, Dr Brown, E. Sakka, A. Kampaite, Dr Wiseman, Dr Valdés-Hernández, Dr Chappell, and Dr Wardlaw contributed to data collection and processing. Dr Stringer, Dr Manning, Dr Morgan, Dr Valdés-Hernández, Dr Chappell, Dr Marshall, Dr Thrippleton, and Dr Wardlaw contributed to data analysis and interpretation. Dr Wardlaw, Dr Stringer, Dr Marshall, and Dr Thrippleton supervised the project. Dr Wardlaw oversaw the work and takes full responsibility for the content. All authors reviewed the article and approved the submitted version.

### Sources of Funding

Dr Sleight is funded by the Medical Research Council (MRC) National Productivity Fund (MR/R502327) and, with A.C.C. Jochems, acknowledges support from the University of Edinburgh College of Medicine and Veterinary Medicine (CMVM). Drs Sleight, Stringer, Arteaga, and Wardlaw are funded by the UK Dementia Research Institute (UKDRI), which receives funding from UKDRI, Ltd, funded by the MRC, Alzheimer’s Society, and Alzheimer’s Research UK. This work received funding from the UKDRI, European Union Horizon 2020 (PHC-03-15, project No. 666881 SVDs@Target), Fondation Leducq Transatlantic Network of Excellence for the Study of Perivascular Spaces in Small Vessel Disease (reference No. 16CVD 05). Furthermore, W. Hewins, Dr Wiseman, and R. Locherty acknowledge funding from Stroke Association (SVD-SOS [Small Vessel Disease - Spotlight On Symptoms], SA PG 19\100068; Stroke Association Postdoctoral Fellowship 18\100026); A.C.C. Jochems from Alzheimer’s Society (ref 486 [AS-CP-18b-001]); Dr Valdés-Hernández, from the Row Fogo Center for Research Into Ageing and the Brain (AD.ROW4.35. BRO-D.FID3668413); Dr Thrippleton from the Scottish Chief Scientist Office through the NHS Lothian Research and Development Office; Dr Clancy from the Chief Scientist Office (CAF/18/08), Princess Margaret Stroke Association Research Development Fellowship and SCREDS (Scottish Clinical Research Excellence Development Scheme) Lectureship Scheme; Dr Arteaga from the Mexican National Council of Science and Technology, Anne Rowling Regenerative Neurology Clinic, and Row Fogo Center for Research Into Small Vessel Diseases; Dr Doubal from The Stroke Association-Garfield Weston Foundation (The Stroke Association Lectureship 2015/04), NHS Research Scotland, and Agnes Parry Endowment at the University of Edinburgh; D.J. Garcia from the Wellcome Trust; Dr Cheng from the China Scholarship Council; and Dr Liu from University of Hong Kong Foundation Postgraduate Fellowship. At the time of contribution, Dr Hamilton was funded by the University of Edinburgh CMVM as part of the Wellcome Trust 4-year PhD in Translational Neuroscience. The 3T research scanner is funded by the Wellcome Trust (104916/Z/14/Z), Dunhill Trust (R380R/1114), Edinburgh and Lothians Health Foundation (2012/17), Muir Maxwell Research Fund, and University of Edinburgh. For the purpose of open access, the author has applied a CC-BY public copyright license to any author accepted manuscript version arising from this submission.

### Disclosures

Drs Morgan and Stringer receive funding from Siemens Healthineers. The other authors report no conflicts.

### Supplemental Material

Figures S1–S2

Tables S1–S9

## Supplementary Material


