# Cerebrovascular reactivity in patients with small vessel disease: a crosssectional study

**DOI:** 10.1161/STROKEAHA.123.042656

**Published:** 2023-10-10

**Authors:** Emilie Sleight, Michael S Stringer, Una Clancy, Carmen Arteaga, Daniela Jaime Garcia, Will Hewins, Angela CC Jochems, Olivia KL Hamilton, Cameron Manning, Alasdair G Morgan, Rachel Locherty, Yajun Cheng, Xiaodi Liu, Junfang Zhang, Iona Hamilton, Charlotte Jardine, Rosalind Brown, Eleni Sakka, Agniete Kampaite, Stewart Wiseman, Maria Valdes-Hernandez, Francesca M Chappell, Fergus N Doubal, Ian Marshall, Michael J Thrippleton, Joanna M Wardlaw

**Affiliations:** 1 Centre for Clinical Brain Sciences, University of Edinburgh, UK; 2 UK Dementia Research Institute, University of Edinburgh, UK; 3 Department of Neurology, West China Hospital of Sichuan University, Chengdu, China; 4 Department of Medicine, University of Hong Kong, Hong Kong; 5 Department of Neurology, Shanghai General Hospital, Shanghai Jiao Tong University School of Medicine, Shanghai, China; 6 Edinburgh Imaging Facility RIE, University of Edinburgh, UK

**Keywords:** Small vessel disease, cerebrovascular reactivity, lacunes, microbleeds, cross-sectional analyses

## Abstract

**Background:**

Cerebrovascular reactivity (CVR) is inversely related to white matter hyperintensity (WMH) severity, a marker of cerebral small vessel disease (SVD). Less is known about the relationship between CVR and other SVD imaging features or cognition. We aimed to investigate these cross-sectional relationships.

**Methods:**

Between 2018 and 2021 in Edinburgh, we recruited patients presenting with lacunar or cortical ischaemic stroke, whom we characterised for SVD features. We measured CVR in subcortical grey matter, normal-appearing white matter (NAWM) and WMH using 3T MRI. We assessed cognition using Montreal Cognitive Assessment (MoCA). Statistical analyses included linear regression models with CVR as outcome, adjusted for age, sex and vascular risk factors. We reported regression coefficients with 95% confidence intervals.

**Results:**

182/208 patients had processable CVR datasets (median age: 68.2 years, 68% male). Although strength of association depended on tissue type, lower CVR in normal-appearing tissues and WMH was associated with larger WMH volume (Bnawm=-0.0073, 95%CI=[-0.0133,-0.0014], %/mmHg per ten-fold increase in %ICV), more lacunes (Bnawm=-0.00129 [-0.00215,-0.00043] %/mmHg per lacune), more microbleeds (Bnawm=-0.00083 [-0.00130,-0.00036] %/mmHg per microbleed), higher deep atrophy score (Bnawm=-0.00218 [-0.00417,-0.00020] %/mmHg per score point increase), higher perivascular space score (Bnawm=-0.0034 [-0.0066,-0.0002] %/mmHg per score point increase in basal ganglia) and higher SVD score (Bnawm=-0.0048 [-0.0075,-0.0021] %/mmHg per score point increase). Lower CVR in normal-appearing tissues was related to lower MoCA without reaching convention statistical significance (Bnawm=0.00065 [-0.00007,0.00137] %/mmHg per score point increase).

**Conclusions:**

Lower CVR in SVD patients was related to more severe SVD burden and worse cognition in this cross-sectional analysis. Longitudinal analysis will help determine if lower CVR predicts worsening SVD severity or vice versa.

**Registration:**

The study is registered ISRCTN12113543 (https://www.isrctn.com/ISRCTN12113543).

## Graphical abstract

**Figure:**
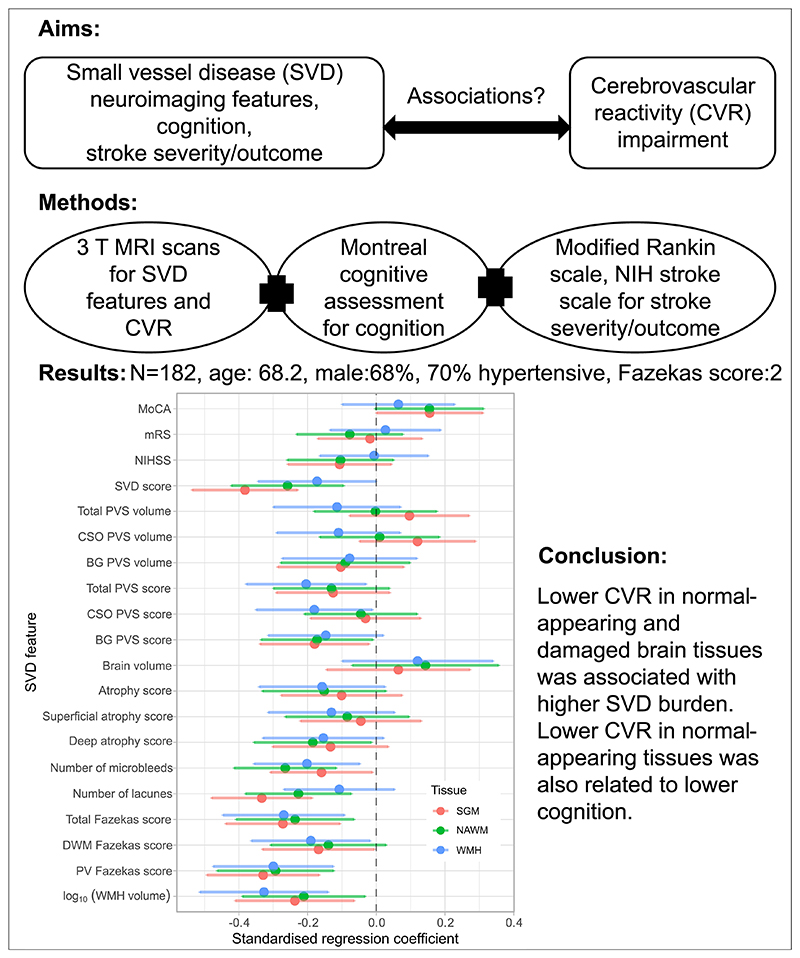

## 1 Introduction

Cerebral small vessel disease (SVD) is a disorder of the cerebral small vessels causing lacunar ischaemic strokes^1^ and vascular cognitive impairment.^2,3^ The associated neuroimaging features observed with magnetic resonance imaging (MRI) are white matter hyperintensities (WMH), lacunes of presumed vascular origin, microbleeds, enlarged perivascular spaces and recent small subcortical infarcts.^4^ Currently, SVD pathophysiology is unclear; no effective treatments are available.^5^ Therefore, identifying vascular dysfunctions and their relationships to disease features and progression may help develop treatments.^6^

One vascular parameter of interest is cerebrovascular reactivity (CVR), which probes the ability of cerebral blood vessels to dilate in response to increased brain demand for energy and is impaired in SVD patients.^7,8,6^ CVR can be obtained by measuring changes in blood oxygen level dependent (BOLD) – an MRI technique sensitive to cerebral blood flow (CBF) and volume (CBV) - in response to a vasodilatory stimulus, including carbon dioxide (CO_2_) enriched air.^7,9^

Previous studies investigating CVR in SVD patients found cross-sectional associations between lower CVR in subcortical grey matter and white matter and higher WMH burden.^8,10,11^ One study noted lower CVR in WMH compared to contralateral normal-appearing white matter.^12^ Furthermore, subcortical grey and white matter CVR are associated with higher blood pressure, but not with global CBF.^8^ White matter CVR is associated with enlarged perivascular spaces in the basal ganglia, increased pulsatility in the venous sinuses and lower cerebrospinal fluid stroke volume in the foramen magnum.^8^ Global CVR reduction is associated with having more microbleeds, but not with the number of lacunes.^13^ Overall, the sample sizes of these studies were relatively small, most of the results have not yet been replicated, and associations of CVR with clinical features such as cognition have not been extensively tested in SVD patients.

Therefore, we aimed to assess CVR in relation to SVD MRI features at 3T, cognition and stroke severity in a large cohort of SVD patients who presented with a minor non-disabling lacunar or cortical ischaemic stroke. We hypothesised that lower CVR in normal-appearing tissues and WMH would be associated with more severe SVD imaging features, worse cognition and stroke severity.

## 2 Methods

We followed the STROBE reporting guidelines.^14^ The data that support the findings of this study will be made available when the study has completed. In the meantime, they are available from the corresponding author upon reasonable request.

### 2.1 Patients

Between August 2018 and June 2021, we recruited patients with mild ischaemic stroke, either lacunar or mild cortical ischaemic stroke, presenting at Edinburgh/Lothian strokes services (Mild Stroke Study 3; ISRCTN 12113543).^15,16^ Mild stroke was defined as a modified Rankin scale (mRS) ≤ 2 and stroke diagnosis was undertaken by specialist stroke physicians and neuroradiologists. We excluded patients with MRI contraindications, major neuronal conditions, severe cardiac and respiratory diseases. All participants gave written informed consent. The Southeast Scotland Regional Ethics Committee approved the study (ref. 18/SS/0044).

Within three months of index stroke, all participants underwent MRI. We recorded medical history and vascular risk factors (VRFs) for each patient and measured blood pressure. We assessed global cognition using the Montreal Cognitive Assessment (MoCA). Stroke severity and degree of patient disability was measured using the NIH stroke scale (NIHSS) and mRS.^17^

### 2.2 MRI acquisitions

The visit included a 1.5h MRI scanning session with breaks for patient comfort. All images were acquired on a 3T MRI scanner (MAGNETOM Prisma, Siemens Healthcare, Erlangen, Germany). We acquired 3D T_1_-weighted (T1W; TR/TE/TI=2500/4.37/1100 ms, flip angle=7°, 1.0 mm^3^ isotropic resolution), 3D T_2_-weighted (T2W; TR/TE=3200/408 ms, 0.9 mm^3^ isotropic resolution), 3D fluid-attenuated inversion recovery (FLAIR; TR/TE/TI=5000/388/1800 ms, 1.0 mm^3^ isotropic resolution), 3D susceptibility-weighted imaging (SWI; TR/TE=28/20 ms, flip angle = 9°, 0.6x0.6x3.0 mm^3^ resolution) images.^15^ We also performed a 2D gradient-echo echo-planar imaging scan to measure CVR (TR/TE=1550/30 ms, flip angle=67°, 2.5 mm^3^ isotropic resolution). Full details of the MRI acquisition protocols including reproducibility can be found in previous works.^7,15,16,18^

During the 12-minute CVR scan, a physician or nurse was present and we administered medical air and 6% CO_2_-enriched air (6:21:73% CO_2_:O_2_:N_2_) alternately for 2 and 3 minutes respectively.^7^ We monitored other physiological parameters: end-tidal CO_2_ (EtCO_2_), end-tidal O_2_, oxygen saturation level, heart and respiration rates.

### 2.3 Analysis of MRI data

Neuroimaging SVD features were assessed using the STRIVE-1 criteria (Table S1).^4^ We visually assessed WMH, separately in periventricular and deep WM, using Fazekas scores. We visually rated perivascular spaces (PVS) score in the basal ganglia (BG) and centrum semiovale (CSO).^19^ We also noted the number of lacunes and microbleeds and rated atrophy in deep and superficial brain areas.^20^ We summed Fazekas, PVS and atrophy scores to get the total Fazekas, PVS and atrophy scores respectively. We computed the SVD score, scoring overall SVD severity.^21^

For each individual, all structural images were co-registered to the subject’s T2W image using FSL FLIRT^22,23^ (FMRIB Software Library, FMRIB Analysis Group, Oxford, United Kingdom). Acute stroke lesions were manually segmented on FLAIR images under supervision of an expert neuroradiologist. WMH were segmented on FLAIR images,^24^ whereas PVS were segmented on T2W images using a previously described computational method.^25,26^ The brain was segmented using the co-registered and combined FLAIR, T1W and T2W images. Normal-appearing white matter (NAWM) masks were generated using an in-house developed processing pipeline that combines FreeSurfer^27,28^ (https://surfer.nmr.mgh.harvard.edu/) and FSL FAST^29^ outputs. Subcortical structures and ventricles were segmented using Freesurfer.^27,28^ All masks were checked and rectified manually if needed. WMH and brain volumes were normalised to the intracranial volume and reported in %ICV units. PVS volumes were normalised to the volume of the region of interest (ROIV) where they were segmented and reported in %ROIV units.

Regarding CVR data processing, subcortical grey matter (SGM) and NAWM masks were eroded in T2W space by 1 mm in all directions to reduce partial volume artefact. To minimise contamination from large blood vessels running along the ventricles, tissue adjacent to the ventricles was excluded using a mask of the ventricles dilated by 5 mm to the left and right, and by 4 mm to the anterior, posterior, superior and inferior directions. We then subtracted the dilated mask from the NAWM and WMH masks. The contribution from other large venous blood vessels was manually removed by comparing all masks to the SWI images. Thereafter, BOLD volumes were temporally realigned. Masks (SGM, NAWM, WMH) were registered to the mean BOLD space and used to compute the mean BOLD signal in each ROI. We used linear regression to model the mean BOLD signal using a time-shifted EtCO_2_ profile and volume number (to account for linear signal drift) as independent variables.^7,18^ We did not model voxel-wise BOLD signals as this lacks robustness against noise.^18^ The optimal delay per subject and ROI was defined as the time-shift of the EtCO_2_ profile that gave lowest sum of squared residuals. CVR (in %/mmHg) was defined as the relative change in BOLD signal per unit change in EtCO_2_. CVR was not assessed in cortical GM due to its thinness, especially in SVD patients where atrophy including cortical thinning is common, and due to large blood vessels running along the brain surface and causing a large ‘blooming’ effect thereby contaminating the cortical signal.^7^

### 2.4 Statistical analysis

Statistical analyses were conducted using R. We modelled CVR separately in SGM, NAWM and WMH. Univariate and multivariable linear regressions were conducted using CVR as outcome and SVD features or cognition as independent variables. In the multivariable analyses, we adjusted the models for age, sex, mean arterial pressure (MAP), smoking history (current/recent versus ex-smoker for more than one year versus never), diagnosis of hypertension, diabetes and hypercholesterolaemia. We checked for collinearity between variables and verified model assumptions: normality of residuals and heteroscedasticity. To ensure normality of residuals, we transformed WMH volumes using the logarithm to the base-10 function.

We excluded missing data from the relevant analyses. We reported coefficients of the linear regressions with 95% confidence intervals (CI) and *p*-values. We did not apply corrections for multiple comparisons as we did not use a significance level. We conducted several sensitivity analyses to verify specific technical points (Table S4-S9).

## 3 Results

**Figure 1:**
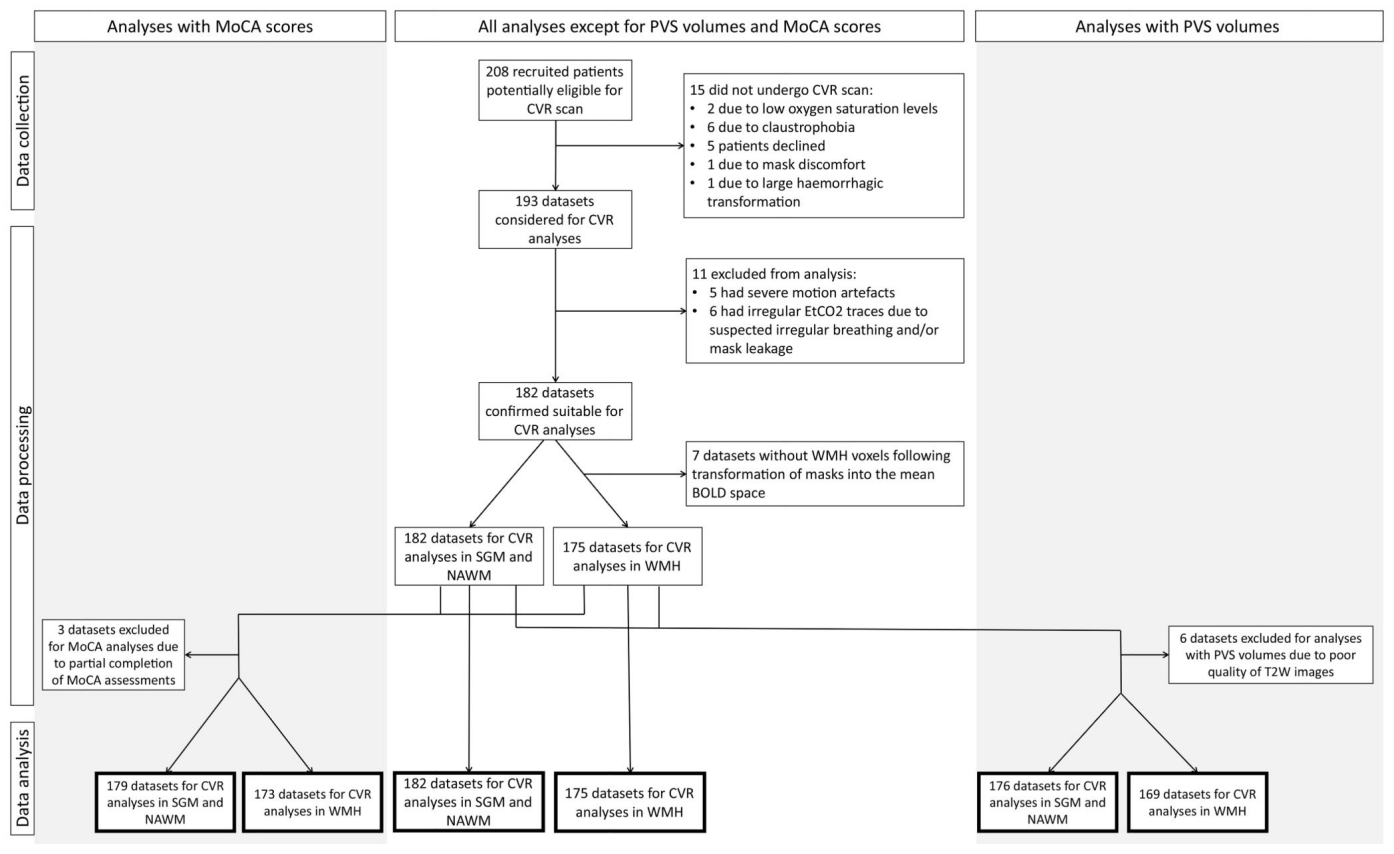
Flow diagram showing data exclusion process prior to the analysis.

We recruited 208 patients of whom 15 did not undergo CVR (Figure 1). We included 182/193 datasets in the analysis (median age: 68.2 years old, 68% male; Table 1). Reasons to exclude 11 datasets are given in Figure 1. Of the remaining 182 datasets, 7 patients did not have WMH voxels following mask registration into the mean BOLD space, thus resulting in 175 datasets specifically for WMH CVR analyses. PVS volumes could not be computed in 6/182 datasets due to poor quality of T2W images. Full MoCA assessment was not available for 3/182 subjects.

CVR was similar in WMH and NAWM (mean inter-region difference [95% CI]: 0.00206 [-0.00379,0.00791] %/mmHg) and highest in SGM (SGM-NAWM CVR difference: 0.128 [0.121,0.134] %/mmHg; Table 1).

**Figure 2:**
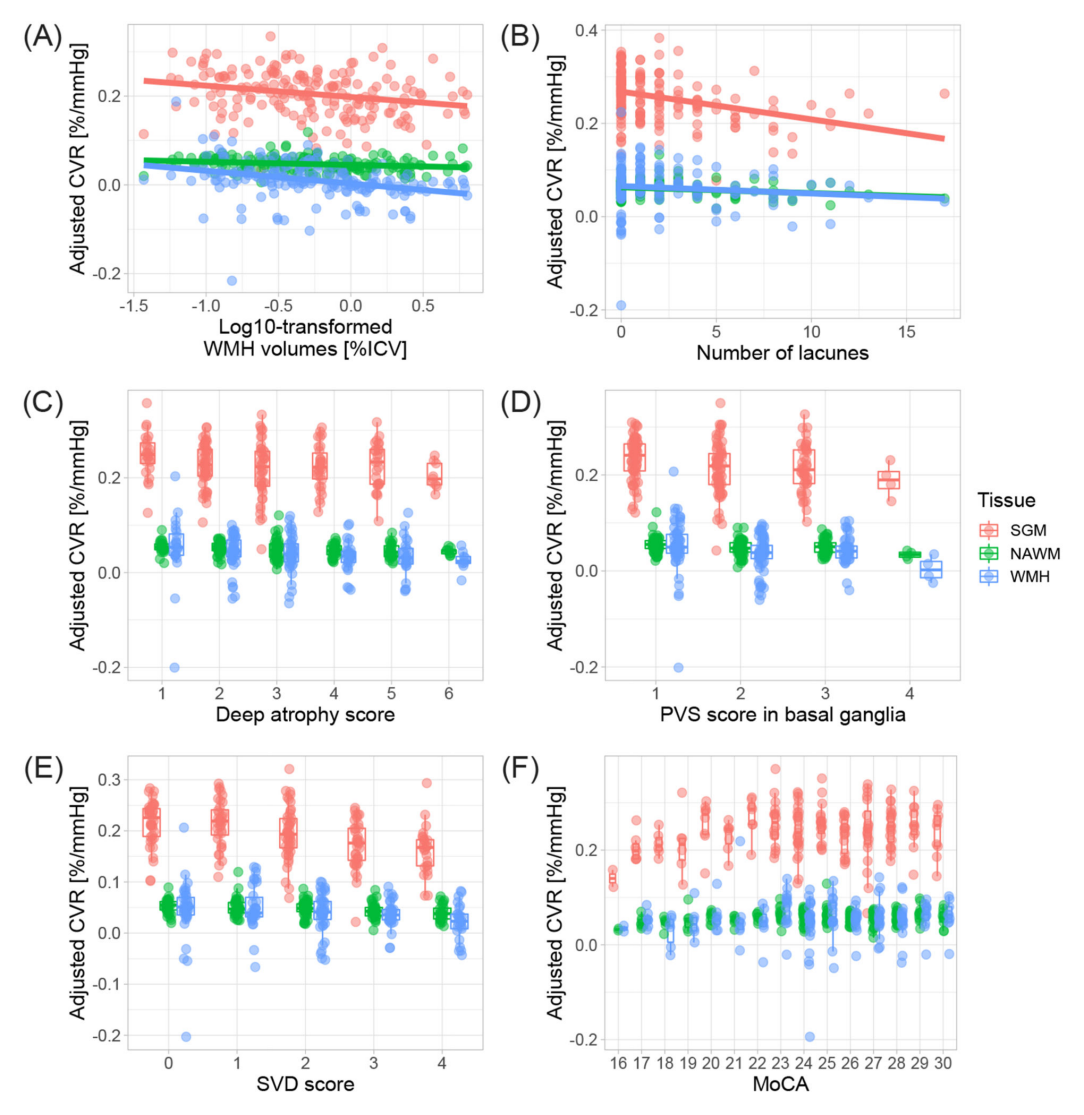
Standardised regression coefficients between SVD features and CVR in SGM (pink), NAWM (green) and WMH (blue). The dots represent the mean standardised coefficients and the horizontal lines the associated 95% confidence intervals. The vertical dashed line emphasises a zero-valued coefficient. Coefficients to left of zero line indicate association with lower CVR. (MoCA: Montreal cognitive assessment, mRS: modified Rankin scale, NIHSS: National Institutes of Health stroke scale, SVD: small vessel disease, PVS: perivascular space, CSO: centrum semiovale, BG: basal ganglia, DWM: deep white matter, PV: periventricular, WMH: white matter hyperintensity, SGM: subcortical grey matter, NAWM: normal-appearing white matter)

**Figure 3:**
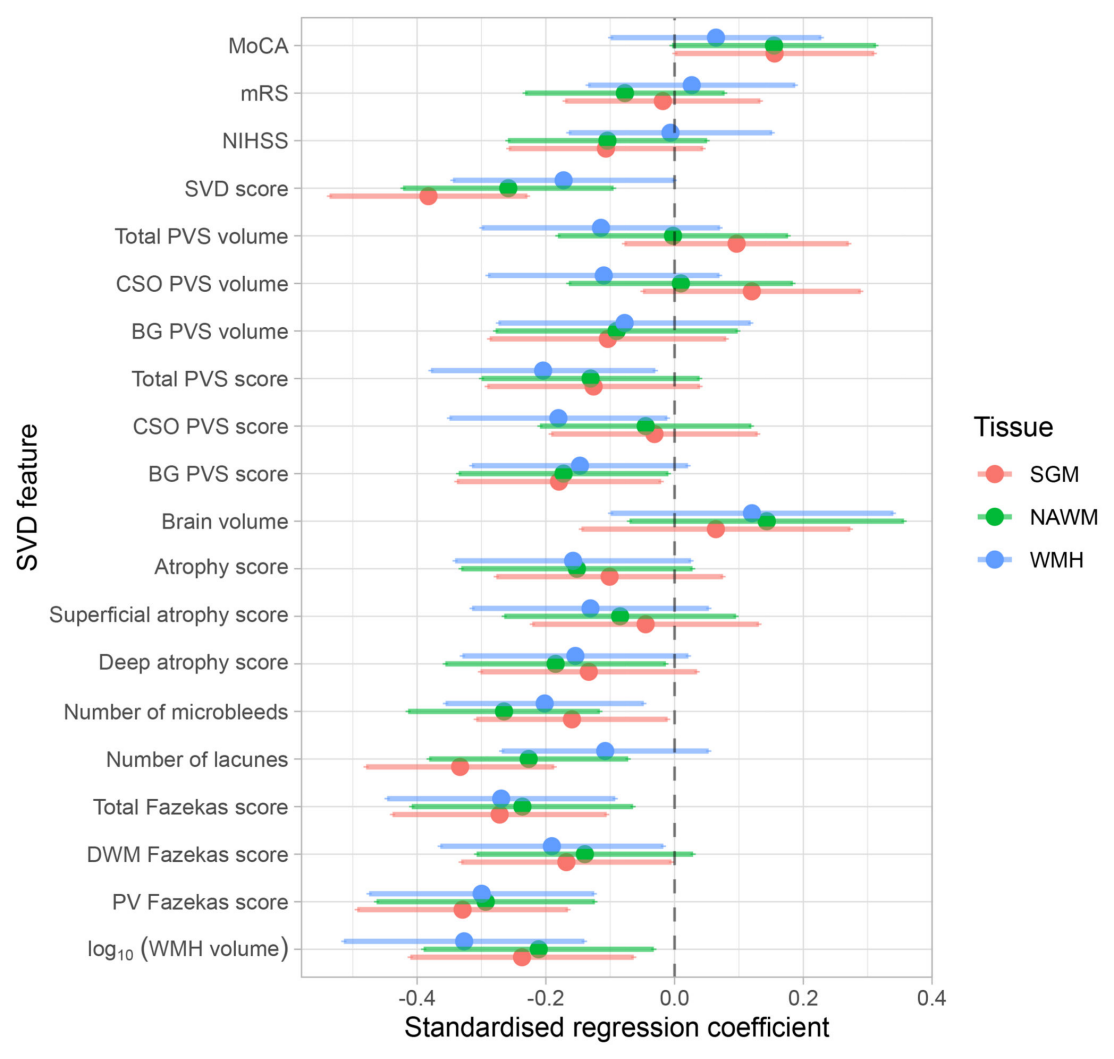
Relationships between adjusted CVR, SVD features and cognition. CVR was adjusted for age, sex and vascular risk factors. The results are shown for adjusted CVR in SGM (pink), NAWM (green) and WMH (blue). In plots A and B, the regression lines are shown. (CVR: cerebrovascular reactivity, WMH: white matter hyperintensity, SVD: small vessel disease, PVS: perivascular space, MoCA: Montreal cognitive assessment, SGM: subcortical grey matter, NAWM: normal-appearing white matter)

Regression coefficients are reported in Table 2 and illustrated in Figures 2 and 3. Lower CVR in most tissues was associated with greater WMH volumes, higher Fazekas scores, more microbleeds, more lacunes and higher SVD scores, although relationships between WMH CVR and lacunes and between NAWM CVR and deep WM Fazekas scores were not conventionally significant. Lower NAWM CVR was associated with higher deep atrophy scores, with a similar relationship for SGM and WMH CVR. We found an association between lower CVR in normal-appearing tissues and higher BG PVS scores, with a similar direction of effect for WMH CVR. Moreover, lower WMH CVR was associated with higher CSO and total PVS scores. There was a general direction of lower CVR in normal-appearing tissues and lower MoCA scores, although not conventionally significant. We did not find associations between CVR, brain volumes, NIHSS and mRS scores.

## 4 Discussion

We investigated how CVR relates to a comprehensive set of SVD features, as well as to cognitive impairment and stroke severity. In this largest study of CVR in SVD to date, CVR was lower in patients with more severe SVD even in normal-appearing tissues, and in association with different SVD features, although the strength of association varied across tissue and lesion types. CVR in normal-appearing tissues and MoCA scores were positively related, although existence of effect did not reach conventional significance. These relationships were independent of age, sex and VRFs. As SVD-related tissue damage accumulates over time,^30^ regions with low CVR could be at risk of deteriorating. Indeed, a previous study (N=45) found that CVR in NAWM that progressed into WMH after one year was lower than in contralateral NAWM.^31^ Future studies should confirm this.

The relationship between lower CVR and higher WMH burden is consistent between WMH volumes and visual scores. Such relationships have been found in previous studies in older subjects with WMH,^12,31–34^ patients with Alzheimer’s disease^35^ and SVD patients with mild stroke.^8^ The effect sizes are similar to those from a previous study.^10^ Overall, the sample size of the current study is larger (N=182 versus N=10-75), thereby making the finding much more robust.

Lower CVR in most ROIs was associated with more lacunes and microbleeds. The coefficient between CVR in WMH and number of lacunes did not reach conventional statistical significance (p<0.05), although the direction of effect is biologically plausible. Two previous studies also investigated those relationships, but found no associations between CVR and number of lacunes.^8,13^ Results for number of microbleeds differed: one study found CVR impairment related to more microbleeds^13^ and the other found no associations.^8^ However, the two studies had much smaller sample sizes (N=49-53), data were acquired at different field strengths (1.5 and 7T) and other brain regions were considered for CVR computation.

Lower NAWM CVR was associated with higher deep atrophy score, whereas other relationships between CVR and brain atrophy did not pass the p<0.05 threshold. However, based on the coefficient and its 95% CIs, one could argue about the existence of an association between lower WMH CVR and higher deep atrophy scores. A previous study^8^ found no associations between CVR and atrophy, possibly due to smaller sample size (N=53).

We found an association between lower CVR in all ROIs and higher BG PVS score with lower confidence in the existence of an effect in the case of CVR in WMH. On the other hand, we found associations between WMH CVR and CSO or total PVS scores. Different relationships with CVR were found when using PVS scores and volumes: whereas scores reflect only a count, volumes will also be influenced by PVS size. Moreover, scores could be limited by floor and ceiling effects.^36^ Previous studies have found that lower CVR^8,37^ and higher vascular pulsatility^38^ are associated with enlarged PVS. Although currently under debate, lower CVR and higher vascular pulsatility could be linked to vascular stiffness, which itself could induce stagnation of interstitial fluid, thereby providing a link between the brain’s waste clearance and vascular systems.^6,39^

We also found an association between lower CVR in all ROIs and higher SVD score in agreement with a previous study.^8^ Therefore, CVR could be a marker reflecting overall SVD severity and should be considered for future clinical studies of SVD.

CVR impairment in normal-appearing tissues was related to worse cognition, although the results did not reach conventional statistical significance. However, this could have been mediated by WMH burden. There were no associations between CVR and stroke outcome or severity, possibly because both were very mild. One previous study also reported no associations between CVR and stroke severity or dependency, though its sample size was smaller.^8^ Previous studies on Alzheimer’s disease have found lower CVR compared to healthy volunteers, but did not report on the relationship between CVR and cognition directly.^35,40^

This work has multiple strengths. We used a reproducible CVR experiment optimised for SVD research.^7,10,18^ Visual assessments of SVD features were systematic, comprehensive and supervised by expert neuroradiologists, and statistical analyses were verified by a professional statistician. Image analysis used pipelines designed and tested in vascular disease. Finally, this is the largest study to date to have assessed CVR impairment in SVD.

There are also some limitations. First, the BOLD contrast is sensitive to CBF, but also to CBV, oxygen extraction fraction, oxygen consumption, haematocrit and vessel morphology; thereby hindering the interpretation of BOLD signal changes. CVR was not assessed in cortical GM due to associated technical challenges, although this would be relevant in future work. Due to limited repeatability,^18^ CVR delay was not investigated in this study. This analysis only included SVD patients with lacunar or cortical stroke, therefore the associations found could differ in other forms of SVD. More males were recruited than females, reflecting male excess in small vessel stroke.^41^ The population had mild stroke, but patients with more severe stroke would not be able to tolerate long scans. Moreover, we only used MoCA to reflect cognition whereas other metrics could be investigated, e.g. Trail making A and B test.^42^ Lastly, this is a cross-sectional study; therefore the relationships found are not causal.

Overall, lower CVR in WMH, NAWM and SGM was associated with SVD burden in patients with mild ischaemic stroke and SVD. The strength of association depended on the tissue and SVD feature type. Further research is needed to understand how CVR impairment relates to the progression of SVD lesions.

## Supplementary Material

Graphical Abstract

STROBE

Supplemental Publication Material

## Figures and Tables

**Table 1:** Population characteristics. Non-binary variables are reported as median [IQR] and binary and smoking variables as number (%).

Variables	Median/number
Age [years]	68.2 [56.4,75.5]
Sex (male, female)	123 (68), 59 (32)
Modified Rankin scale	1 [1,1]
NIHSS	1 [0,2]
Systolic blood pressure [mmHg]	147 [136,161]
Diastolic blood pressure [mmHg]	85 [76,92]
Mean arterial pressure [mmHg]	105 [98,114]
Diabetes diagnosis	36 (20)
Hypertension diagnosis	128 (70)
Hypercholesterolaemia diagnosis	134 (74)
Smoker (current, ever, never)	28 (15), 70 (39), 84 (46)
Stroke type (lacunar, cortical)	77 (42), 108 (58)
WMH volume [ml]	8.09 [3.76,18.77]
WMH volume [%ICV]	0.51 [0.24,1.14]
Number of lacunes	1 [0,3]
Number of microbleeds	0 [0,0]
Deep atrophy score	3 [2,4]
Superficial atrophy score	3 [2,4]
Total atrophy score	6 [4,8]
Brain volume [ml]	1075 [1005,1176]
Brain volume [%ICV]	67.3 [64.6, 71.0]
Periventricular Fazekas score	1 [1,2]
Deep WM Fazekas score	1 [1,2]
Total Fazekas score	3 [2,4]
BG PVS score	2 [1,3]
CSO PVS score	2 [2,3]
Total PVS score	4 [3,5]
BG PVS volume [ml]	2.8 [2.0,3.9]
BG PVS volume [%ROIV]	4.9 [3.3,6.4]
CSO PVS volume [ml]	10.8 [6.6,16.6]
CSO PVS volume [%ROIV]	3.3 [2.0,5.6]
Total PVS volume [ml]	13.8 [8.8,20.1]
Total PVS volume [%ROIV]	3.6 [2.2,5.7]
SVD score	1 [0,2]
Montreal cognitive assessment	25 [22,27]
Subcortical grey matter CVR [%/mmHg]	0.171 [0.135,0.207]
Normal-appearing WM CVR [%/mmHg]	0.042 [0.033,0.054]
WMH CVR [%/mmHg]	0.040 [0.025,0.064]

Abbreviations - NIHSS: National Institutes of Health stroke scale, ICV: intracranial volume, BG: basal ganglia, PVS: perivascular space, CSO: centrum semiovale, ROIV: volume of region of interest, SVD: small vessel disease, CVR: cerebrovascular reactivity, WM: white matter, WMH: WM hyperintensity

**Table 2:** Adjusted analyses. Each row represents a different statistical model where the SVD predictor of interest is given in the first column. The associated regression coefficient B, its 95% confidence interval and p-value are given in columns 2-4. The last column gives the units of B. All models were corrected for age, sex and vascular risk factors.

Variables	SGM CVR	NAWM CVR	WMH CVR	Units of B
WMH volume [%ICV]	B=-0.0254 [-0.0440, -0.0067] p=0.008	B=-0.0073 [-0.0133, -0.0014] p=0.016	B=-0.0287 [-0.0451, -0.0122] p=0.001	%BOLD/mmHg per log_1_0(%ICV)
Periventricular Fazekas score	B=-0.0202 [-0.0303, -0.0100] p<0.001	B=-0.0057 [-0.0090, -0.0025] p=0.001	B=-0.0151 [-0.0239, -0.0062] p=0.001	%BOLD/mmHg per score unit
Deep WM Fazekas score	B=-0.0108 [-0.0214, -0.0001] p=0.048	B=-0.0029 [-0.0063, 0.0005] p=0.092	B=-0.0101 [-0.0194, -0.0008] p=0.033	%BOLD/mmHg per score unit
Total Fazekas score	B=-0.00899 [-0.01457, -0.00341] p=0.002	B=-0.00252 [-0.00431, -0.00073] p=0.006	B=-0.00734 [-0.01220, -0.00247] p=0.003	%BOLD/mmHg per score unit
Number of lacunes	B=-0.00590 [-0.00852, -0.00328] p<0.001	B=-0.00129 [-0.00215, -0.00043] p=0.003	B=-0.00154 [-0.00389, 0.00082] p=0.199	%BOLD/mmHg per lacune
Number of microbleeds	B=-0.00159 [-0.00310, -0.00008] p=0.039	B=-0.00083 [-0.00130, -0.00036] p=0.001	B=-0.00167 [-0.00295, -0.00039] p=0.011	%BOLD/mmHg per microbleed
Deep atrophy score	B=-0.00526 [-0.01151, 0.00099] p=0.098	B=-0.00218 [-0.00417, -0.00020] p=0.031	B=-0.00482 [-0.01016, 0.00052] p=0.077	%BOLD/mmHg per score unit
Superficial atrophy score	B=-0.00194 [-0.00853, 0.00464] p=0.561	B=-0.00106 [-0.00316, 0.00103] p=0.317	B=-0.00410 [-0.00973, 0.00154] p=0.153	%BOLD/mmHg per score unit
Total atrophy score	B=-0.00215 [-0.00562, 0.00132] p=0.222	B=-0.00097 [-0.00207, 0.00014] p=0.085	B=-0.00261 [-0.00557, 0.00035] p=0.083	%BOLD/mmHg per score unit
Brain volume [%ICV]	B=0.000734 [-0.001655, 0.003122] p=0.545	B=0.000507 [-0.000248, 0.001263] p=0.187	B=0.00113 [-0.00093, 0.00318] p=0.281	%BOLD/mmHg per %ICV
BG PVS score	B=-0.0109 [-0.0210, -0.0009] p=0.034	B=-0.0034 [-0.0066, -0.0002] p=0.039	B=-0.0074 [-0.0162, 0.0013] p=0.094	%BOLD/mmHg per score unit
CSO PVS score	B=-0.00138 [-0.01067, 0.00790] p=0.769	B=-0.00082 [-0.00378, 0.00214] p=0.585	B=-0.00840 [-0.01642, -0.00038] p=0.040	%BOLD/mmHg per score unit
Total PVS score	B=-0.00409 [-0.00986, 0.00169] p=0.165	B=-0.00142 [-0.00326, 0.00042] p=0.130	B=-0.00573 [-0.01071, -0.00074] p=0.025	%BOLD/mmHg per score unit
BG PVS volume [%ROIV]	B=0.00036 [-0.00256, 0.00328] p=0.808	B=-0.00005 [-0.00098, 0.00088] p=0.911	B=0.00145 [-0.00107, 0.00398] p=0.257	%BOLD/mmHg per %ROIV
CSO PVS volume [%ROIV]	B=0.00036 [-0.00222, 0.00294] p=0.782	B=0.00012 [-0.00070, 0.00094] p=0.769	B=0.00087 [-0.00134, 0.00308] p=0.439	%BOLD/mmHg per %ROIV
Total PVS volume [%ROIV]	B=0.00038 [-0.00232, 0.00307] p=0.783	B=0.00011 [-0.00074, 0.00097] p=0.794	B=0.00101 [-0.00130, 0.00332] p=0.390	%BOLD/mmHg per %ROIV
SVD score	B=-0.0157 [-0.0241, -0.0073] p<0.001	B=-0.0048 [-0.0075, -0.0021] p=0.001	B=-0.0139 [-0.0211, -0.0067] p<0.001	%BOLD/mmHg per score unit
NIHSS	B=-0.00401 [-0.00985, 0.00182] p=0.176	B=-0.00133 [-0.00318, 0.00053] p=0.161	B=-0.00014 [-0.00513, 0.00486] p=0.957	%BOLD/mmHg per score unit
Modified Rankin scale	B=-0.00150 [-0.01336, 0.01036] p=0.803	B=-0.00210 [-0.00587, 0.00167] p=0.274	B=0.00173 [-0.00858, 0.01203] p=0.741	%BOLD/mmHg per score unit
Montreal cognitive assessment	B=0.00220 [-0.00005, 0.00445] p=0.056	B=0.00065 [-0.00007, 0.00137] p=0.076	B=0.00044 [-0.00152, 0.00240] p=0.660	%BOLD/mmHg per score unit

Abbreviations - ICV: intracranial volume, NIHSS: National Institutes of Health stroke scale, BG: basal ganglia, PVS: perivascular space, CSO: centrum semiovale, ROIV: volume of region of interest, SVD: small vessel disease, CVR: cerebrovascular reactivity, SGM: subcortical grey matter, WM: white matter, NAWM: normal-appearing WM, WMH: WM hyperintensity.

## Abbreviations

BG: basal ganglia
BOLD: blood oxygen level dependent
CBF: cerebral blood flow
CBV: cerebral blood volume
CI: confidence interval
CO_2_: carbon-dioxide
CSO: centrum semiovale
CVR: cerebrovascular reactivity
EtCO_2_: end-tidal carbon-dioxide
MAP: mean arterial pressure
MoCA: Montreal cognitive assessment
MRI: magnetic resonance imaging
mRS: modified Rankin scale
NAWM: normal-appearing white matter
NIHSS: national institute of health stroke scale
PVS: perivascular space
ROI: region of interest
ROIV: volume of region of interest
SGM: subcortical grey matter
SVS: small vessel disease
VRFs: vascular risk factors
WMH: white matter hyperintensity
