# Supplementary material for: Cerebrovascular reactivity in patients with small vessel disease: a crosssectional study
Source: Stroke. Author manuscript; Available in PMC 2023 Oct 22. (PMC10589433; doi:10.1161/STROKEAHA.123.042656)
Supplement: Supplemental Publication Material [file EMS188308-supplement-Supplemental_Publication_Material.pdf]

SUPPLEMENTARY MATERIAL

1 Visual scores

Table S1. Range and meaning of visual scores

Scores	Range	Meaning of upper bound
Periventricular Fazekas, Deep white matter Fazekas	0−3	highest WMH burden
Total Fazekas	0−6	highest WMH burden
Basal ganglia PVS, Centrum semiovale PVS	0−4	> 40 enlarged PVS per side
Total PVS	0−8	> 80 enlarged PVS per side
Superficial atrophy, Deep atrophy	0−6	severe atrophy
Total atrophy	0−12	severe atrophy
SVD	0−4	all SVD features present (WMH, lacunes, microbleeds and PVS)
NIHSS	0−42	Worse stroke severity
Modified Rankin scale	0−5	Higher degree of disability
MoCA	0−30	Better global cognition

2 CVR data quality

CVR was extracted from the SGM, NAWM and WMH masks (Figure S1). The median [IQR] number of voxels remaining in the mean BOLD space was 765 [687, 861], 6628 [5278, 8002] and 43 [14, 209] for SGM, NAWM and WMH respectively.

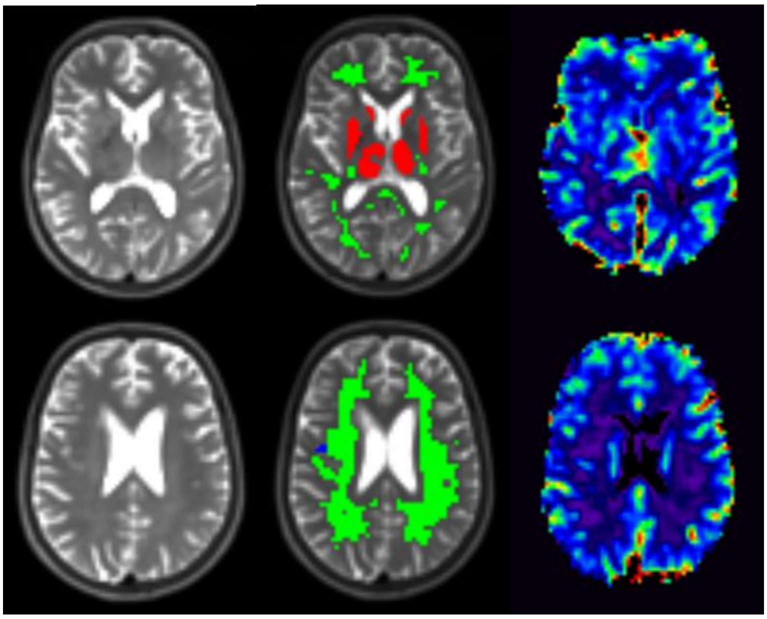

Figure S1. Example of tissue masks used for CVR analysis. Two slices of the T2W image of a patient co-registered to the mean BOLD image are shown in the first column. The masks overlapped on the co-registered T2W image are shown in the second column: CVR was extracted from the SGM (red), NAWM (green) and WMH (blue) tissues. The related CVR maps are displayed in the third column. The latter were spatially smoothed for visual purposes using a Gaussian filter with full width at half maximum of 4 mm.

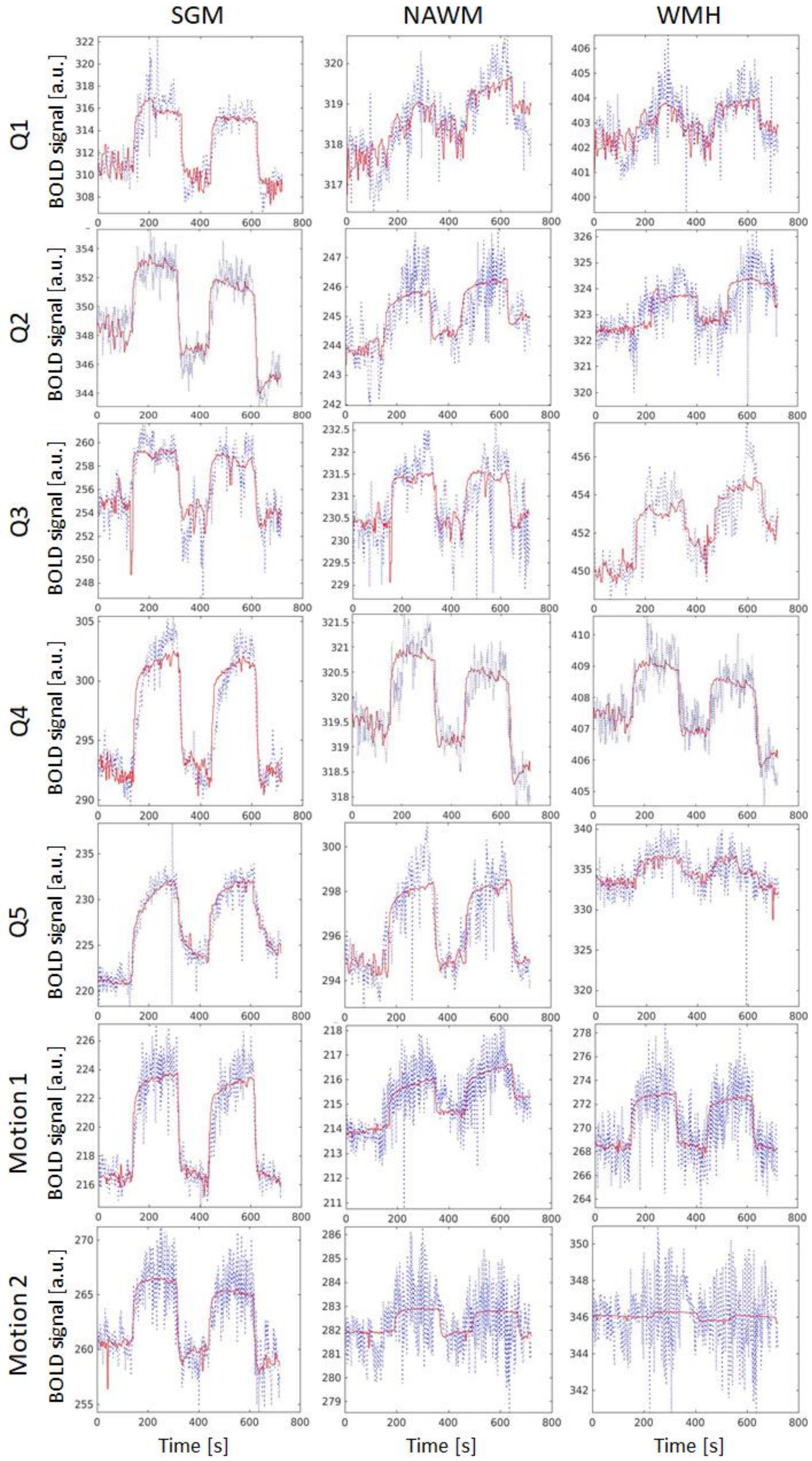

Figure S3. Examples of BOLD signal fits in SGM (1st column), NAWM (2nd column) and WMH (3rd column). Rows 1-5 give examples of BOLD signal modelling as a function of CVR quintile (Q1-Q5). Rows 6-7 give examples of BOLD signal modelling where the mean framewise displacement was high (>0.70) and were

excluded in the sensitivity analysis related to motion. The blue dots represent the BOLD timepoints and the red curves the fits.

3 R packages

Statistical analyses were conducted using R (version 3.6.1; cran.r-project.org/) with the additional packages: tidyverse, ggplot2, insight, sjPlot and car.

4 Supplementary statistical analyses

4.1 Univariate analyses

Table S2. Regression coefficients from univariate analyses. Each row represents a different statistical model where the SVD predictor of interest is given in the first column. The associated regression coefficient B, its 95% confidence interval and p-value are given in columns 2-4. The last column gives the units of B. (NIHSS: National Institutes of Health stroke scale, mRS: modified Rankin scale, BG: basal ganglia, PVS: perivascular space, CSO: centrum semiovale, ROIV: volume of region of interest, SVD: small vessel disease, CVR: cerebrovascular reactivity, SGM: subcortical grey matter, NAWM: normal-appearing white matter, WMH: white matter hyperintensity)

Variables	SGM CVR	NAWM CVR	WMH CVR	Units of B
WMH volume [%ICV]	B=-0.0323 [-0.0474, -0.0172] p<0.001	B=-0.00733 [-0.01212, -0.00254] p=0.003	B=-0.0241 [-0.0372, -0.0110] p<0.001	%BOLD/mmHg per log_{10}(%ICV)
Periventricular Fazekas score	B=-0.0222 [-0.0307, -0.0138] p<0.001	B=-0.00522 [-0.00792, -0.00251] p<0.001	B=-0.0131 [-0.0203, -0.0058] p<0.001	%BOLD/mmHg per score unit
Deep white matter Fazekas score	B=-0.0162 [-0.0255, -0.0069] p=0.001	B=-0.00354 [-0.00647, -0.00062] p=0.018	B=-0.0104 [-0.0182, -0.0026] p=0.009	%BOLD/mmHg per score unit
Total Fazekas score	B=-0.0108 [-0.0154, -0.0061] p<0.001	B=-0.00246 [-0.00394, -0.00098] p=0.001	B=-0.00658 [-0.01053, -0.00262] p=0.001	%BOLD/mmHg per score unit
Number of lacunes	B=-0.00564 [-0.00813, -0.00315] p<0.001	B=-0.00112 [-0.00192, -0.00033] p=0.006	B=-0.00148 [-0.00363, 0.00068] p=0.179	%BOLD/mmHg per lacune

Number of microbleeds	B=-0.00122 [-0.00270, 0.00027] p=0.108	B=-0.000666 [-0.001119, -0.000213] p=0.004	B=-0.00133 [-0.00255, -0.00012] p=0.032	%BOLD/mmHg per microbleed
Deep atrophy score	B=-0.00624 [-0.01164, -0.00085] p=0.024	B=-0.00202 [-0.00369, -0.00035] p=0.018	B=-0.00470 [-0.00920, -0.00020] p=0.041	%BOLD/mmHg per score unit
Superficial atrophy score	B=-0.00483 [-0.01031, 0.00065] p=0.084	B=-0.00134 [-0.00304, 0.00036] p=0.122	B=-0.00429 [-0.00884, 0.00027] p=0.065	%BOLD/mmHg per score unit
Total atrophy score	B=-0.00310 [-0.00597, -0.00023] p=0.035	B=-0.000940 [-0.001830, -0.000050] p=0.039	B=-0.00251 [-0.00490, -0.00012] p=0.040	%BOLD/mmHg per score unit
Brain volume [%ICV]	B=0.00185 [0.00019, 0.00351] p=0.029	B=0.000523 [0.000007, 0.001038] p=0.047	B=0.00119 [-0.00021, 0.00259] p=0.094	%BOLD/mmHg per %ICV
BG PVS score	B=-0.0130 [-0.0221, -0.0039] p=0.005	B=-0.00337 [-0.00621, -0.00054] p=0.020	B=-0.00738 [-0.01509, 0.00032] p=0.060	%BOLD/mmHg per score unit
CSO PVS score	B=-0.00580 [-0.01425, 0.00264] p=0.177	B=-0.00151 [-0.00413, 0.00111] p=0.256	B=-0.00820 [-0.01529, -0.00111] p=0.024	%BOLD/mmHg per score unit
Total PVS score	B=-0.00604 [-0.01108, -0.00101] p=0.019	B=-0.00157 [-0.00314, -0.00000] p=0.050	B=-0.00526 [-0.00952, -0.00100] p=0.016	%BOLD/mmHg per score unit
BG PVS volume [%ROIV]	B=-0.00410 [-0.00750, -0.00071] p=0.018	B=-0.000834 [-0.001889, 0.000222] p=0.121	B=-0.00178 [-0.00463, 0.00106] p=0.218	%BOLD/mmHg per %ROIV
CSO PVS volume [%ROIV]	B=-0.000639 [-0.003846, 0.002569] p=0.695	B=-0.000410 [-0.001396, 0.000576] p=0.413	B=-0.00227 [-0.00490, 0.00036] p=0.090	%BOLD/mmHg per %ROIV

Total PVS volume [%ROIV]	B=-0.00125 [-0.00461, 0.00212] p=0.466	B=-0.000503 [-0.001538, 0.000532] p=0.339	B=-0.00239 [-0.00516, 0.00037] p=0.090	%BOLD/mmHg per %ROIV
SVD score	B=-0.0156 [-0.0209, -0.0103] p<0.001	B=-0.00312 [-0.00485, -0.00139] p<0.001	B=-0.00551 [-0.01025, -0.00076] p=0.023	%BOLD/mmHg per score unit
NIHSS	B=-0.00577 [-0.01139, -0.00016] p=0.044	B=-0.00145 [-0.00320, 0.00029] p=0.102	B=-0.000675 [-0.005355, 0.004004] p=0.776	%BOLD/mmHg per score unit
Modified Rankin scale	B=-0.00446 [-0.01600, 0.00708] p=0.447	B=-0.00232 [-0.00589, 0.00124] p=0.200	B=0.000040 [-0.009663, 0.009743] p=0.994	%BOLD/mmHg per score unit
MoCA	B=0.00325 [0.00108, 0.00541] p=0.003	B=0.000821 [0.000147, 0.001496] p=0.017	B=0.00111 [-0.00070, 0.00292] p=0.226	%BOLD/mmHg per score unit

4.2 Regression coefficients for age and patient demographics

Table S3. Cross-sectional linear regression between CVR and WMH volume, adjusted for age, sex and vascular risk factors. Columns 2 to 4 contain the regression coefficients, 95% confidence intervals and p-values. The last column gives the units of the regression coefficient. (CVR: cerebrovascular reactivity, SGM: subcortical grey matter, NAWM: normal-appearing white matter, WMH: white matter hyperintensity, MAP: mean arterial pressure)

Variables	SGM CVR	NAWM CVR	WMH CVR	Units of B
WMH volume	B=-0.0256 [-0.0443, -0.0068] p=0.008	B=-0.00705 [-0.01302, -0.00108] p=0.021	B=-0.0288 [-0.0453, -0.0124] p=0.001	%/mmHg per $\log_{10}$(%ICV)
Age	B=-0.000533 [-0.001325, 0.000258] p=0.185	B=-0.000083 [-0.000335, 0.000170] p=0.519	B=0.000077 [-0.000614, 0.000769] p=0.825	%/mmHg per year
Sex [Male]	B=0.0104 [-0.0062, 0.0270] p=0.218	B=0.00218 [-0.00311, 0.00748] p=0.417	B=0.000601 [-0.013438, 0.014641] p=0.933	%/mmHg
MAP	B=-0.000055 [-0.000657, 0.000547] p=0.857	B=-0.000027 [-0.000219, 0.000165] p=0.782	B=0.000173 [-0.000343, 0.000690] p=0.509	%/mmHg per mmHg
Diabetes	B=-0.00191 [-0.02143, 0.01761] p=0.847	B=-0.00167 [-0.00790, 0.00456] p=0.598	B=-0.00205 [-0.01874, 0.01464] p=0.809	%/mmHg
Smoker [Ever]	B=-0.0116 [-0.0282, 0.0049] p=0.168	B=-0.00193 [-0.00722, 0.00335] p=0.471	B=-0.00272 [-0.01681, 0.01136] p=0.703	%/mmHg
Smoker [Current]	B=-0.0195 [-0.0420, 0.0030] p=0.088	B=-0.00349 [-0.01067, 0.00368] p=0.338	B=-0.00259 [-0.02179, 0.01661] p=0.790	%/mmHg
Hypertension	B=0.00869 [-0.00936, 0.02673] p=0.343	B=0.00466 [-0.00110, 0.01042] p=0.112	B=0.0128 [-0.0028, 0.0284] p=0.107	%/mmHg
Hypercholesterolaemia	B=0.00255	B=0.00166	B=-0.000728	%/mmHg

	[-0.01455, 0.01965] p=0.769	[-0.00380, 0.00712] p=0.549	[-0.015344, 0.013888] p=0.922

4.3 Sensitivity analyses

4.3.1 Adjusting for WMH volume

Table S4. Regression coefficients after adjusting for WMH volume. Each row represents a different statistical model where the SVD predictor of interest is given in the first column. The associated regression coefficient B, its 95% confidence interval and p-value are given in columns 2-4. The last column gives the units of B. All models were corrected for WMH volume, age, sex, MAP, diagnosis of diabetes, hypertension, and hypercholesterolaemia and history of smoking. (NIHSS: National Institutes of Health stroke scale, mRS: modified Rankin scale, BG: basal ganglia, PVS: perivascular space, CSO: centrum semiovale, ROIV: volume of region of interest, SVD: small vessel disease, CVR: cerebrovascular reactivity, SGM: subcortical grey matter, NAWM: normal-appearing white matter, WMH: white matter hyperintensity)

Variables	SGM CVR	NAWM CVR	WMH CVR	Units of B
Number of lacunes	B=-0.00535 [-0.00825, -0.00246] p<0.001	B=-0.00100 [-0.00195, -0.00005] p=0.039	B=0.000191 [-0.002343, 0.002725] p=0.882	%BOLD/mmHg per lacune
Number of microbleeds	B=-0.00101 [-0.00262, 0.00059] p=0.214	B=-0.000726 [-0.001229, -0.000223] p=0.005	B=-0.00101 [-0.00236, 0.00034] p=0.141	%BOLD/mmHg per microbleed
Deep atrophy score	B=-0.00308 [-0.00945, 0.00330] p=0.342	B=-0.00166 [-0.00369, 0.00036] p=0.107	B=-0.00242 [-0.00783, 0.00299] p=0.379	%BOLD/mmHg per score unit
Superficial atrophy score	B=-0.000470 [-0.007035, 0.006095] p=0.888	B=-0.000658 [-0.002751, 0.001435] p=0.536	B=-0.00287 [-0.00840, 0.00266] p=0.307	%BOLD/mmHg per score unit
Total atrophy score	B=-0.00106 [-0.00457, 0.00244] p=0.550	B=-0.000690 [-0.001806, 0.000426] p=0.224	B=-0.00154 [-0.00449, 0.00141] p=0.305	%BOLD/mmHg per score unit

Brain volume [%ICV]	B=0.000011 [-0.002400, 0.002422] p=0.993	B=0.000324 [-0.000444, 0.001092] p=0.406	B=0.000399 [-0.001643, 0.002441] p=0.700	%BOLD/mmHg per %ICV
BG PVS score	B=-0.00694 [-0.01785, 0.00396] p=0.211	B=-0.00221 [-0.00569, 0.00127] p=0.211	B=-0.00185 [-0.01115, 0.00745] p=0.695	%BOLD/mmHg per score unit
CSO PVS score	B=0.00169 [-0.00776, 0.01114] p=0.724	B=0.000137 [-0.002880, 0.003154] p=0.929	B=-0.00521 [-0.01331, 0.00289] p=0.206	%BOLD/mmHg per score unit
Total PVS score	B=-0.00150 [-0.00770, 0.00470] p=0.634	B=-0.000651 [-0.002630, 0.001327] p=0.517	B=-0.00284 [-0.00815, 0.00247] p=0.293	%BOLD/mmHg per score unit
BG PVS volume [%ROIV]	B=-0.000202 [-0.004823, 0.004418] p=0.931	B=0.000094 [-0.001369, 0.001557] p=0.899	B=0.00151 [-0.00241, 0.00543] p=0.447	%BOLD/mmHg per %ROIV
CSO PVS volume [%ROIV]	B=0.00413 [0.00046, 0.00780] p=0.028	B=0.000507 [-0.000669, 0.001683] p=0.396	B=-0.000455 [-0.003614, 0.002703] p=0.776	%BOLD/mmHg per %ROIV
Total PVS volume [%ROIV]	B=0.00412 [0.00010, 0.00814] p=0.045	B=0.000534 [-0.000753, 0.001820] p=0.414	B=-0.000270 [-0.003729, 0.003189] p=0.878	%BOLD/mmHg per %ROIV
NIHSS	B=-0.00311 [-0.00890, 0.00268] p=0.291	B=-0.000970 [-0.002819, 0.000878] p=0.302	B=0.000972 [-0.003901, 0.005846] p=0.694	%BOLD/mmHg per score unit
Modified Rankin scale	B=-0.000139 [-0.011888, 0.011610] p=0.981	B=-0.00153 [-0.00527, 0.00221] p=0.420	B=0.00320 [-0.00684, 0.01324] p=0.530	%BOLD/mmHg per score unit

MoCA	B=0.00192 [-0.00038, 0.00422] p=0.100	B=0.000609 [-0.000124, 0.001341] p=0.103	B=0.000341 [-0.001607, 0.002288] p=0.730	%BOLD/mmHg per score unit

4.3.2 Adjusting for $EtCO_2$ baseline

Table S5. Regression coefficients after adjusting for $EtCO_2$ baseline. Each row represents a different statistical model where the SVD predictor of interest is given in the first column. The associated regression coefficient B, its 95% confidence interval and p-value are given in columns 2-4. The last column gives the units of B. All models were corrected for $EtCO_2$ baseline, age, sex, MAP, diagnosis of diabetes, hypertension, and hypercholesterolaemia and history of smoking. (ICV: intracranial volume, NIHSS: National Institutes of Health stroke scale, mRS: modified Rankin scale, BG: basal ganglia, PVS: perivascular space, CSO: centrum semiovale, ROIV: volume of region of interest, SVD: small vessel disease, CVR: cerebrovascular reactivity, SGM: subcortical grey matter, NAWM: normal-appearing white matter, WMH: white matter hyperintensity)

Variables	SGM CVR	NAWM CVR	WMH CVR	Units of B
Log_{10}-transformed WMH volume [log_{10}(%ICV)]	B=-0.0220 [-0.0398, -0.0042] p=0.016	B=-0.00614 [-0.01198, -0.00029] p=0.040	B=-0.0278 [-0.0445, -0.0110] p=0.001	%BOLD/mmHg per log_{10}(%ICV)
Periventricular Fazekas score	B=-0.0173 [-0.0269, -0.0076] p=0.001	B=-0.00485 [-0.00804, -0.00167] p=0.003	B=-0.0142 [-0.0232, -0.0053] p=0.002	%BOLD/mmHg per score unit
Deep white matter Fazekas score	B=-0.00879 [-0.01892, 0.00134] p=0.089	B=-0.00225 [-0.00557, 0.00106] p=0.182	B=-0.00936 [-0.01871, -0.00001] p=0.050	%BOLD/mmHg per score unit
Total Fazekas score	B=-0.00759 [-0.01290, -0.00228] p=0.005	B=-0.00207 [-0.00382, -0.00033] p=0.020	B=-0.00689 [-0.01181, -0.00196] p=0.006	%BOLD/mmHg per score unit
Number of lacunes	B=-0.00531 [-0.00778, -0.00284] p<0.001	B=-0.00109 [-0.00193, -0.00026] p=0.010	B=-0.00135 [-0.00371, 0.00101] p=0.260	%BOLD/mmHg per lacune
Number of microbleeds	B=-0.00172 [-0.00313,	B=-0.000859 [-0.001310,	B=-0.00170 [-0.00298,	%BOLD/mmHg per microbleed

	-0.00030]<br>p=0.018	-0.000408]<br>p<0.001	-0.00041]<br>p=0.010	
Deep atrophy score	B=-0.00421<br>[-0.01010,<br>0.00169]<br>p=0.161	B=-0.00195<br>[-0.00386,<br>-0.00003]<br>p=0.046	B=-0.00442<br>[-0.00976,<br>0.00093]<br>p=0.104	%BOLD/mmHg per score unit
Superficial atrophy score	B=-0.00124<br>[-0.00745,<br>0.00497]<br>p=0.694	B=-<br>0.000878<br>[-0.002903,<br>0.001147]<br>p=0.393	B=-0.00387<br>[-0.00951,<br>0.00177]<br>p=0.177	%BOLD/mmHg per score unit
Total atrophy score	B=-0.00164<br>[-0.00491,<br>0.00164]<br>p=0.325	B=-<br>0.000841<br>[-0.001905,<br>0.000223]<br>p=0.120	B=-0.00243<br>[-0.00539,<br>0.00053]<br>p=0.107	%BOLD/mmHg per score unit
Brain volume [%ICV]	B=0.00103<br>[-0.00121,<br>0.00328]<br>p=0.364	B=0.000582<br>[-0.000147,<br>0.001311]<br>p=0.117	B=0.00120<br>[-0.00085,<br>0.00326]<br>p=0.248	%BOLD/mmHg per %ICV
BG PVS score	B=-0.0109<br>[-0.0203,<br>-0.0015]<br>p=0.023	B=-0.00327<br>[-0.00634,<br>-0.00019]<br>p=0.038	B=-0.00740<br>[-0.01606,<br>0.00126]<br>p=0.093	%BOLD/mmHg per score unit
CSO PVS score	B=-<br>0.000484<br>[-0.009197,<br>0.008230]<br>p=0.913	B=-<br>0.000486<br>[-0.003330,<br>0.002359]<br>p=0.736	B=-0.00803<br>[-0.01603,<br>-0.00002]<br>p=0.049	%BOLD/mmHg per score unit
Total PVS score	B=-0.00372<br>[-0.00913,<br>0.00169]<br>p=0.176	B=-0.00125<br>[-0.00301,<br>0.00052]<br>p=0.165	B=-0.00555<br>[-0.01051,<br>-0.00060]<br>p=0.028	%BOLD/mmHg per score unit
BG PVS volume [%ROIV]	B=-0.00216<br>[-0.00611,<br>0.00180]<br>p=0.283	B=-<br>0.000564<br>[-0.001846,<br>0.000718]<br>p=0.386	B=-0.00129<br>[-0.00497,<br>0.00239]<br>p=0.489	%BOLD/mmHg per %ROIV
CSO PVS volume [%ROIV]	B=0.00301<br>[-0.00038,	B=0.000203<br>[-0.000904,	B=-0.00170<br>[-0.00486,	%BOLD/mmHg per %ROIV

	0.00640]<br>p=0.081	0.001309]<br>p=0.718	0.00145]<br>p=0.288	
Total PVS volume [%ROIV]	B=0.00263<br>[-0.00104,<br>0.00629]<br>p=0.159	B=0.000127<br>[-0.001067,<br>0.001321]<br>p=0.834	B=-0.00186<br>[-0.00527,<br>0.00155]<br>p=0.282	%BOLD/mmHg per %ROIV
SVD score	B=-0.0134<br>[-0.0192, -<br>0.0077]<br>p<0.001	B=-0.00274<br>[-0.00468,<br>-0.00079]<br>p=0.006	B=-0.00496<br>[-0.01053,<br>0.00061]<br>p=0.081	%BOLD/mmHg per score unit
NIHSS	B=-0.00208<br>[-0.00766,<br>0.00350]<br>p=0.463	B=-<br>0.000735<br>[-0.002557,<br>0.001086]<br>p=0.426	B=0.000585<br>[-0.004473,<br>0.005644]<br>p=0.820	%BOLD/mmHg per score unit
Modified Rankin scale	B=0.000476<br>[-0.010757,<br>0.011708]<br>p=0.933	B=-0.00140<br>[-0.00506,<br>0.00226]<br>p=0.451	B=0.00265<br>[-0.00772,<br>0.01302]<br>p=0.615	%BOLD/mmHg per score unit
MoCA	B=0.00202<br>[-0.00017,<br>0.00421]<br>p=0.071	B=0.000639<br>[-0.000075,<br>0.001353]<br>p=0.079	B=0.000631<br>[-0.001382,<br>0.002644]<br>p=0.537	%BOLD/mmHg per score unit

4.3.3 Excluding datasets with high motion during CVR-BOLD scan

Table S6. Regression coefficients after excluding datasets with high motion during CVR-BOLD scan. Each row represents a different statistical model where the SVD predictor of interest is given in the first column. The associated regression coefficient B, its 95% confidence interval and p-value are given in columns 2-4. The last column gives the units of B. All models were corrected for age, sex, MAP, diagnosis of diabetes, hypertension, and hypercholesterolaemia and history of smoking. A total of 12 datasets was removed. (ICV: intracranial volume, NIHSS: National Institutes of Health stroke scale, mRS: modified Rankin scale, BG: basal ganglia, PVS: perivascular space, CSO: centrum semiovale, ROIV: volume of region of interest, SVD: small vessel disease, CVR: cerebrovascular reactivity, SGM: subcortical grey matter, NAWM: normal-appearing white matter, WMH: white matter hyperintensity)

Variables	SGM CVR	NAWM CVR	WMH CVR	Units of B
Log_{10}-transformed WMH volume [log_{10}(%ICV)]	B=-0.0195 [-0.0392, 0.0002] p=0.052	B=-0.00599 [-0.01219, 0.00021] p=0.058	B=-0.0267 [-0.0440, -0.0093] p=0.003	%BOLD/mm Hg per log_{10}(%ICV)
Periventricular Fazekas score	B=-0.0176 [-0.0284, -0.0068] p=0.002	B=-0.00500 [-0.00843, -0.00158] p=0.004	B=-0.0138 [-0.0233, -0.0044] p=0.004	%BOLD/mm Hg per score unit
Deep white matter Fazekas score	B=-0.00845 [-0.01941, 0.00251] p=0.130	B=-0.00224 [-0.00570, 0.00122] p=0.202	B=-0.00879 [-0.01830, 0.00072] p=0.070	%BOLD/mm Hg per score unit
Total Fazekas score	B=-0.00747 [-0.01331, -0.00163] p=0.012	B=-0.00208 [-0.00393, -0.00023] p=0.028	B=-0.00654 [-0.01162, -0.00146] p=0.012	%BOLD/mm Hg per score unit
Number of lacunes	B=-0.00534 [-0.00819, -0.00249] p<0.001	B=-0.00120 [-0.00212, -0.00028] p=0.011	B=-0.00142 [-0.00396, 0.00111] p=0.269	%BOLD/mm Hg per lacune

Number of microbleeds	B=-0.00140 [-0.00296, 0.00015] p=0.076	B=-0.000813 [-0.001291, -0.000336] p=0.001	B=-0.00144 [-0.00277, -0.00012] p=0.033	%BOLD/mm Hg per microbleed
Deep atrophy score	B=-0.00381 [-0.01031, 0.00269] p=0.249	B=-0.00210 [-0.00413, -0.00007] p=0.042	B=-0.00443 [-0.00997, 0.00112] p=0.117	%BOLD/mm Hg per score unit
Superficial atrophy score	B=-0.00124 [-0.00813, 0.00565] p=0.722	B=-0.000962 [-0.003128, 0.001203] p=0.381	B=-0.00358 [-0.00947, 0.00238] p=0.232	%BOLD/mm Hg per score unit
Total atrophy score	B=-0.00152 [-0.00513, 0.00210] p=0.409	B=-0.000912 [-0.002043, 0.000219] p=0.113	B=-0.00234 [-0.00541, 0.00074] p=0.135	%BOLD/mm Hg per score unit
Brain volume [%ICV]	B=-0.000163 [-0.002843, 0.002518] p=0.905	B=0.000722 [-0.000115, 0.001558] p=0.090	B=0.00119 [-0.00112, 0.00350] p=0.309	%BOLD/mm Hg per %ICV
BG PVS score	B=-0.0101 [-0.0204, 0.0001] p=0.052	B=-0.00292 [-0.00615, 0.00032] p=0.077	B=-0.00740 [-0.01626, 0.00147] p=0.101	%BOLD/mm Hg per score unit
CSO PVS score	B=0.000783 [-0.008831, 0.010396] p=0.872	B=-0.000179 [-0.003206, 0.002849] p=0.907	B=-0.00656 [-0.01494, 0.00181] p=0.124	%BOLD/mm Hg per score unit
Total PVS score	B=-0.00299 [-0.00888, 0.00290] p=0.317	B=-0.00101 [-0.00286, 0.00084] p=0.283	B=-0.00490 [-0.00999, 0.00019] p=0.059	%BOLD/mm Hg per score unit
BG PVS volume [%ROIV]	B=-0.00131 [-0.00569, 0.00307] p=0.557	B=-0.000376 [-0.001747, 0.000996] p=0.589	B=-0.00141 [-0.00521, 0.00239] p=0.465	%BOLD/mm Hg per %ROIV

CSO PVS volume [%ROIV]	B=0.00387 [0.00011, 0.00763] p=0.044	B=0.000539 [-0.000652, 0.001730] p=0.373	B=-0.00148 [-0.00477, 0.00181] p=0.376	%BOLD/mm Hg per %ROIV
Total PVS volume [%ROIV]	B=0.00359 [-0.00049, 0.00766] p=0.084	B=0.00048 [-0.00080, 0.00177] p=0.461	B=-0.00167 [-0.00523, 0.00189] p=0.355	%BOLD/mm Hg per %ROIV
SVD score	B=-0.0141 [-0.0205, -0.0076] p<0.001	B=-0.00301 [-0.00511, -0.00091] p=0.005	B=-0.00533 [-0.01121, 0.00055] p=0.075	%BOLD/mm Hg per score unit
NIHSS	B=-0.00467 [-0.01105, 0.00171] p=0.150	B=-0.00234 [-0.00433, -0.00035] p=0.021	B=-0.00156 [-0.00702, 0.00391] p=0.575	%BOLD/mm Hg per score unit
Modified Rankin scale	B=0.00109 [-0.01146, 0.01365] p=0.864	B=-0.00149 [-0.00543, 0.00246] p=0.458	B=0.00316 [-0.00776, 0.01407] p=0.568	%BOLD/mm Hg per score unit
MoCA	B=0.00240 [-0.00002, 0.00483] p=0.052	B=0.000934 [0.000177, 0.001690] p=0.016	B=0.000932 [-0.001167, 0.003031] p=0.382	%BOLD/mm Hg per score unit

4.3.4 Excluding datasets with short hypercapnic paradigm

Table S7. Regression coefficients after excluding datasets with short hypercapnic paradigm. Each row represents a different statistical model where the SVD predictor of interest is given in the first column. The associated regression coefficient B, its 95% confidence interval and p-value are given in columns 2-4. The last column gives the units of B. All models were corrected for age, sex, MAP, diagnosis of diabetes, hypertension, and hypercholesterolaemia and history of smoking. A total of 15 datasets (15/182 for SGM, NAWM CVR analyses and 15/175 for WMH CVR) was removed. (NIHSS: National Institutes of Health stroke scale, mRS: modified Rankin scale, ICV: intracranial volume, BG: basal ganglia, PVS: perivascular space, CSO: centrum semiovale, ROIV: volume of region of interest, SVD: small vessel disease, CVR: cerebrovascular reactivity, SGM: subcortical grey matter, NAWM: normal-appearing white matter, WMH: white matter hyperintensity)

Variables	SGM CVR	NAWM CVR	WMH CVR	Units of B
Log_{10}-transformed WMH volume [log_{10}(%ICV)]	B=-0.0305 [-0.0504, -0.010] p=0.003	B=-0.00678 [-0.01275, -0.00081] p=0.026	B=-0.0210 [-0.0355, -0.0065] p=0.005	%BOLD/mm Hg per log_{10}(%ICV)
Periventricular Fazekas score	B=-0.0230 [-0.0337, -0.0123] p<0.001	B=-0.00501 [-0.00826, -0.00177] p=0.003	B=-0.00935 [-0.01712, -0.00158] p=0.019	%BOLD/mm Hg per score unit
Deep white matter Fazekas score	B=-0.0110 [-0.0222, 0.0001] p=0.053	B=-0.00241 [-0.00574, 0.00092] p=0.155	B=-0.00934 [-0.01716, -0.00152] p=0.020	%BOLD/mm Hg per score unit
Total Fazekas score	B=-0.00983 [-0.01569, -0.00396] p=0.001	B=-0.00214 [-0.00391, -0.00038] p=0.018	B=-0.00541 [-0.00959, -0.00123] p=0.012	%BOLD/mm Hg per score unit
Number of lacunes	B=-0.00618 [-0.00888, -0.00349] p<0.001	B=-0.00126 [-0.00208, -0.00043] p=0.003	B=-0.00203 [-0.00399, -0.00007] p=0.043	%BOLD/mm Hg per lacune
Number of microbleeds	B=-0.00163 [-0.00319, -0.00007] p=0.040	B=-0.000756 [-0.001210, -0.000303] p=0.001	B=-0.00181 [-0.00286, -0.00076] p=0.001	%BOLD/mm Hg per microbleed

Deep atrophy score	B=-0.00546 [-0.01218, 0.00126] p=0.111	B=-0.00259 [-0.00455, -0.00062] p=0.010	B=-0.00540 [-0.01001, -0.00079] p=0.022	%BOLD/mm Hg per score unit
Superficial atrophy score	B=-0.00221 [-0.00914, 0.00472] p=0.529	B=-0.00158 [-0.00362, 0.00046] p=0.128	B=-0.00376 [-0.00855, 0.00103] p=0.123	%BOLD/mm Hg per score unit
Total atrophy score	B=-0.00226 [-0.00595, 0.00143] p=0.227	B=-0.00123 [-0.00231, -0.00014] p=0.027	B=-0.00268 [-0.00521, -0.00015] p=0.038	%BOLD/mm Hg per score unit
Brain volume [%ICV]	B=0.00118 [-0.00133, 0.00369] p=0.354	B=0.000573 [-0.000167, 0.001314] p=0.128	B=0.000758 [-0.000994, 0.002510] p=0.394	%BOLD/mm Hg per %ICV
BG PVS score	B=-0.0146 [-0.0252, -0.0040] p=0.007	B=-0.00317 [-0.00634, 0.00000] p=0.050	B=-0.00764 [-0.01510, -0.00018] p=0.045	%BOLD/mm Hg per score unit
CSO PVS score	B=-0.00220 [-0.01191, 0.00752] p=0.656	B=-0.000100 [-0.002983, 0.002783] p=0.946	B=-0.00909 [-0.01582, -0.00236] p=0.008	%BOLD/mm Hg per score unit
Total PVS score	B=-0.00548 [-0.01151, 0.00055] p=0.075	B=-0.00104 [-0.00284, 0.00076] p=0.254	B=-0.00602 [-0.01021, -0.00183] p=0.005	%BOLD/mm Hg per score unit
BG PVS volume [%ROIV]	B=-0.00336 [-0.00785, 0.00114] p=0.142	B=-0.00104 [-0.00237, 0.00028] p=0.122	B=-0.00165 [-0.00482, 0.00153] p=0.307	%BOLD/mm Hg per %ROIV
CSO PVS volume [%ROIV]	B=0.00225 [-0.00152, 0.00603] p=0.240	B=0.000045 [-0.001075, 0.001166] p=0.936	B=-0.00119 [-0.00384, 0.00146] p=0.377	%BOLD/mm Hg per %ROIV
Total PVS volume [%ROIV]	B=0.00174 [-0.00236, 0.00584] p=0.402	B= -0.000082 [-0.001295, 0.001131] p=0.894	B=-0.00140 [-0.00427, 0.00148] p=0.338	%BOLD/mm Hg per %ROIV
SVD score	B=0.00434 [-0.00923, 0.01790] p=0.529	B= -0.000563	B=-0.00380 [-0.01356, 0.00596] p=0.443	%BOLD/mm Hg per score unit

		[-0.004572, 0.003446] p=0.782		
NIHSS	B=0.00676 [-0.00314, 0.01666] p=0.179	B=0.00133 [-0.00160, 0.00427] p=0.370	B=-0.00412 [-0.01121, 0.00297] p=0.252	%BOLD/mm Hg per score unit
Modified Rankin scale	B=0.00698 [-0.00365, 0.01761] p=0.197	B=0.00125 [-0.00190, 0.00440] p=0.436	B=-0.00444 [-0.01207, 0.00318] p=0.251	%BOLD/mm Hg per score unit
MoCA	B=-0.0168 [-0.0231, -0.0105] p<0.001	B=-0.00297 [-0.00494, -0.00100] p=0.003	B=-0.00531 [-0.01003, -0.00058] p=0.028	%BOLD/mm Hg per score unit

4.3.5 Excluding datasets where masks contain low number of voxels in mean BOLD space

Table S8. Regression coefficients after excluding datasets where masks contain low number of voxels in mean BOLD space. Each row represents a different statistical model where the SVD predictor of interest is given in the first column. The associated regression coefficient B, its 95% confidence interval and p-value are given in the second column. The last column gives the units of B. All models were corrected for age, sex, MAP, diagnosis of diabetes, hypertension, and hypercholesterolaemia and history of smoking. Only WMH CVR analysis was affected by this sensitivity analysis where 34 datasets were removed. (ICV: intracranial volume, NIHSS: National Institutes of Health stroke scale, mRS: modified Rankin scale, BG: basal ganglia, PVS: perivascular space, CSO: centrum semiovale, ROIV: volume of region of interest, SVD: small vessel disease, CVR: cerebrovascular reactivity, WMH: white matter hyperintensity)

Variables	WMH CVR	Units of B
Log_{10}-transformed WMH volume [log_{10}(%ICV)]	B=-0.0233 [-0.0395, -0.0071] p=0.005	%BOLD/mm Hg per log_{10}(%ICV)
Periventricular Fazekas score	B=-0.0143 [-0.0220, -0.0066] p<0.001	%BOLD/mm Hg per score unit
Deep white matter Fazekas score	B=-0.00864 [-0.01651, -0.00076] p=0.032	%BOLD/mm Hg per score unit
Total Fazekas score	B=-0.00673 [-0.01094, -0.00253] p=0.002	%BOLD/mm Hg per score unit
Number of lacunes	B=-0.00193 [-0.00383, -0.00002] p=0.047	%BOLD/mm Hg per lacune

Number of microbleeds	B=-0.00190 [-0.00289, -0.00090] p<0.001	%BOLD/mm Hg per microbleed
Deep atrophy score	B=-0.00348 [-0.00811, 0.00116] p=0.140	%BOLD/mm Hg per score unit
Superficial atrophy score	B=-0.00176 [-0.00655, 0.00303] p=0.468	%BOLD/mm Hg per score unit
Total atrophy score	B=-0.00153 [-0.00407, 0.00100] p=0.234	%BOLD/mm Hg per score unit
Brain volume [%ICV]	B=0.00101 [-0.00073, 0.00275] p=0.253	%BOLD/mm Hg per %ICV
BG PVS score	B=-0.00377 [-0.01128, 0.00374] p=0.322	%BOLD/mm Hg per score unit
CSO PVS score	B=-0.00303 [-0.01037, 0.00432] p=0.416	%BOLD/mm Hg per score unit
Total PVS score	B=-0.00256 [-0.00711, 0.00200] p=0.268	%BOLD/mm Hg per score unit
BG PVS volume [%ROIV]	B=-0.000677 [-0.003754, 0.002400] p=0.664	%BOLD/mm Hg per %ROIV
CSO PVS volume [%ROIV]	B=-0.000272 [-0.002899,	%BOLD/mm Hg per %ROIV

	0.002355] p=0.838	
Total PVS volume [%ROIV]	B=-0.000354 [-0.003198, 0.002489] p=0.806	%BOLD/mmHg per %ROIV
SVD score	B=-0.00395 [-0.00878, 0.00089] p=0.109	%BOLD/mmHg per score unit
NIHSS	B=-0.000376 [-0.004591, 0.003838] p=0.860	%BOLD/mmHg per score unit
Modified Rankin scale	B=0.000292 [-0.008773, 0.009357] p=0.949	%BOLD/mmHg per score unit
MoCA	B=0.000891 [-0.000828, 0.002611] p=0.307	%BOLD/mmHg per score unit

4.3.6 Excluding datasets with structural images of poorer quality

Table S8. Regression coefficients after excluding datasets with structural images of poorer quality. Each row represents a different statistical model where the SVD predictor of interest is given in the first column. The associated regression coefficient B, its 95% confidence interval and p-value are given in columns 2-4. The last column gives the units of B. All models were corrected for age, sex, MAP, diagnosis of diabetes, hypertension, and hypercholesterolaemia and history of smoking. A total of 3 WMH volumes, 3 PVS volumes (BG and CSO included) and 2 brain volumes were removed. (ICV: intracranial volume, BG: basal ganglia, PVS: perivascular space, CSO: centrum semiovale, ROIV: volume of region of interest, CVR: cerebrovascular reactivity, SGM: subcortical grey matter, NAWM: normal-appearing white matter, WMH: white matter hyperintensity)

Variables	GM CVR	NAWM CVR	WMH CVR	Units of B
Log_{10}-transformed WMH volume [log_{10}(%ICV)]	B=-0.0293 [-0.0482, -0.0104] p=0.003	B=-0.00790 [-0.01397, -0.00184] p=0.011	B=-0.0286 [-0.0451, -0.0120] p=0.001	%BOLD/mm Hg per log_{10}(%ICV)
Brain volume [%ICV]	B=0.000642 [-0.001801, 0.003086] p=0.604	B=0.000460 [-0.000316, 0.001236] p=0.244	B=0.00116 [-0.00096, 0.00328] p=0.282	%BOLD/mm Hg per %ICV
BG PVS volume [%ROIV]	B=-0.00280 [-0.00728, 0.00167] p=0.218	B=-0.000359 [-0.001775, 0.001058] p=0.618	B=-0.00111 [-0.00504, 0.00282] p=0.578	%BOLD/mm Hg per %ROIV
CSO PVS volume [%ROIV]	B=0.00256 [-0.00123, 0.00635] p=0.184	B=0.000358 [-0.000842, 0.001558] p=0.556	B=-0.00172 [-0.00503, 0.00159] p=0.306	%BOLD/mm Hg per %ROIV
Total PVS volume [%ROIV]	B=0.00214 [-0.00199, 0.00626] p=0.308	B=0.000315 [-0.000989, 0.001618] p=0.634	B=-0.00187 [-0.00547, 0.00173] p=0.305	%BOLD/mm Hg per %ROIV
